# Hippo/MST blocks breast cancer by downregulating WBP2 oncogene expression via miRNA processor Dicer

**DOI:** 10.1038/s41419-020-02901-3

**Published:** 2020-08-21

**Authors:** Shen Kiat Lim, Hossein Tabatabaeian, Ssu Yi Lu, Shin-Ae Kang, Gopinath Meenakshi Sundaram, Prabha Sampath, Siew Wee Chan, Wan Jin Hong, Yoon Pin Lim

**Affiliations:** 1 Department of Biochemistry, Yong Loo Lin School of Medicine, National University of Singapore, Singapore, 117545 Singapore; 2 Cancer Science Institute of Singapore, National University of Singapore, Singapore, 117599 Singapore; 3 Skin Research Institute of Singapore, Agency for Science Technology and Research, Singapore, 138648 Singapore; 4 Program in Cancer and Stem Cell Biology, Duke-NUS Graduate Medical School, Singapore, 169857 Singapore; 5 Institute of Molecular and Cell Biology, Agency for Science Technology and Research, Singapore, 138673 Singapore; 6 National University Cancer Institute, Singapore, 119082 Singapore; 7 NUS Graduate School for Integrative Sciences and Engineering, National University of Singapore, Singapore, 117456 Singapore

**Keywords:** Breast cancer, miRNAs

## Abstract

WBP2 transcription coactivator is an emerging oncoprotein and a key node of convergence between EGF and Wnt signaling pathways. Understanding how WBP2 is regulated has important implications for cancer therapy. WBP2 is tightly controlled by post-translational modifications, including phosphorylation and ubiquitination, leading to changes in subcellular localization, protein–protein interactions, and protein turnover. As the function of WBP2 is intricately linked to YAP and TAZ, we hypothesize that WBP2 is negatively regulated by the Hippo tumor suppressor pathway. Indeed, MST is demonstrated to negatively regulate WBP2 expression in a kinase-dependent but LATS-independent manner. This was observed in the majority of the breast cancer cell lines tested. The effect of MST was enhanced by SAV and concomitant with the inhibition of the transcription co-activation, in vitro and in vivo tumorigenesis activities of WBP2, resulting in good prognosis in xenografts. Downregulation of WBP2 by MST involved miRNA but not proteasomal or lysosomal degradation. Our data support the existence of a novel MST-Dicer signaling axis, which in turn regulates both WBP2 CDS- and UTR-targeting miRNAs expression, including miR-23a. MiR-23a targets the 3′UTR of WBP2 mRNA directly. Significant inverse relationships between WBP2 and MST or miR23a expression levels in clinical specimens were observed. In conclusion, WBP2 is a target of the Hippo/MST kinase; MST is identified as yet another rheostat in the regulation of WBP2 and its oncogenic function. The findings have implications in targeted therapeutics and precision medicine for breast cancer.

## Introduction

The Hippo tumor suppressor pathway is an evolutionarily conserved signaling cascade that controls organ development in Drosophila by inhibiting cell proliferation, promoting apoptosis, regulating cell fates, and limiting cell size^1–4^. Its core kinase cascade comprises MST & LATS serine/threonine kinases and their regulatory proteins, SAV and MOB. Activation of the Hippo pathway results in the phosphorylation and sequestration of YAP and TAZ in the cytosol. This prevents them from activating the oncogenic TEAD transcription factors in the nucleus. Inactivation of the Hippo pathway concomitant with the activation of YAP and/or TAZ promote in vitro transformation and in vivo tumorigenesis^1,5–7^.

WW domain-Binding Protein 2 (WBP2) was first identified as a cognate ligand of the WW domain of YAP^8,9^. WBP2’s transcription co-activator function is important to cancer^10^. Binding of WBP2 to E6AP potentiated Estrogen and Progesterone Receptor co-activation^11^ by binding to the phosphorylated form of RNA polymerase II and histone acetyltransferase p300^12^. WBP2 cooperated with Drosophila YAP (Yki) to drive tissue growth in Drosophila^13^ and was required for the oncogenic property of TAZ in breast cancer (BC)^14^. WBP2 was overexpressed in >75% of clinical BC cases. Patients with higher WBP2 expression had poorer overall and disease-free survival^15^. WBP2 is increasingly implicated in other cancers, such as skin, brain, and liver^10^.

WBP2 expression and activity are tightly controlled. Tyrosine phosphorylation of WBP2 promotes its nucleus entry, protein–protein interaction, and transcription co-activation activity^16^. In contrast, ubiquitination and proteasomal degradation limits WBP2 oncogenic function^15^. As a member of a functional network comprising YAP/TAZ, we hypothesize WBP2 to be negatively regulated by the Hippo pathway. Here, WBP2 is shown to be regulated by yet another distinct mechanism involving MST, Dicer and miRNA.

## Results

### Hippo/MST negatively regulates WBP2’s expression and function

**Fig. 1:**
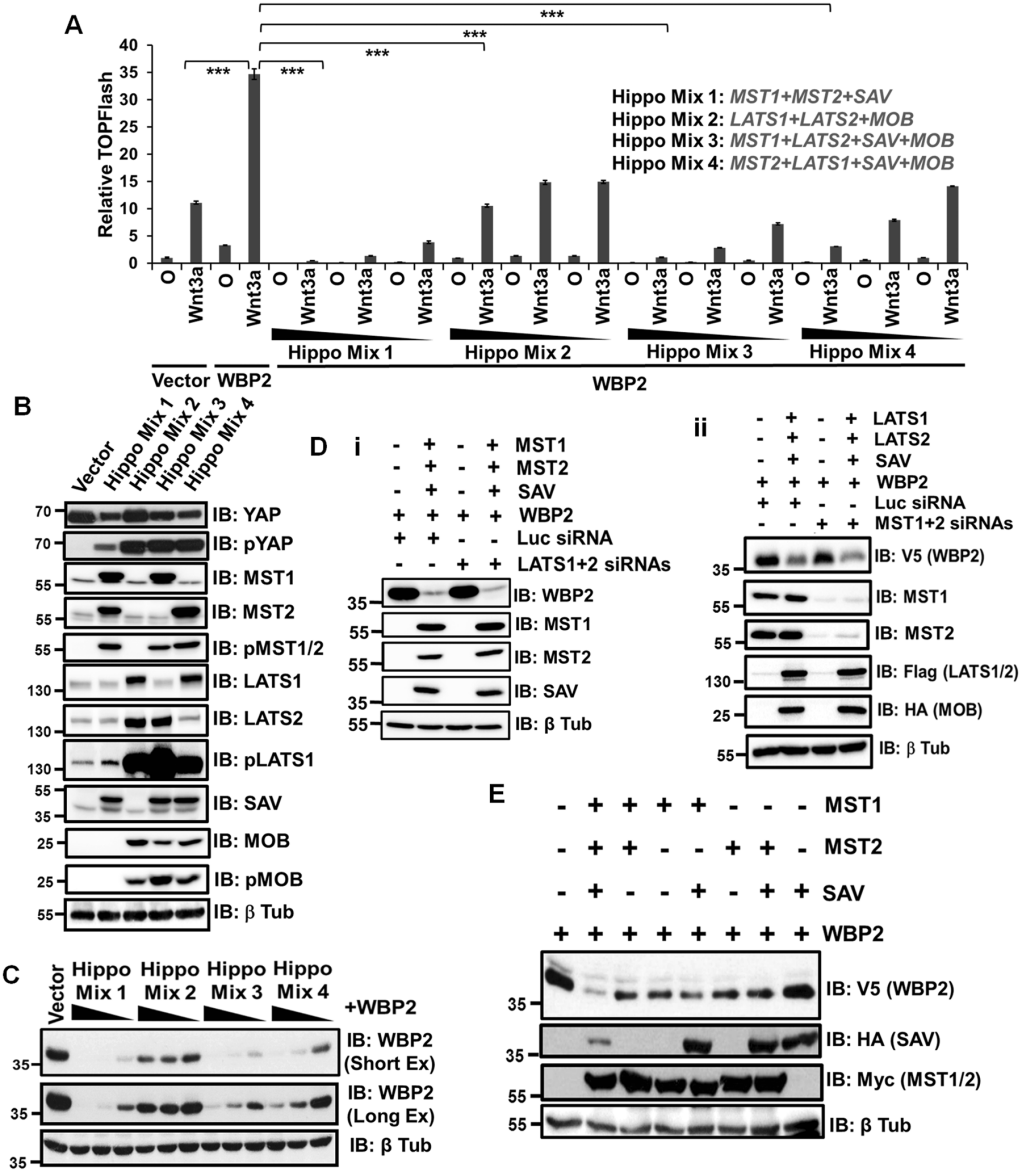
Hippo/MST Negatively Regulates WBP2’s Expression and Function. **a** Overexpression of MST and/or LATS kinase in combination with their regulatory proteins SAV and MOB abolished the WNT3A-induced, WBP2-mediated TCF reporter activity in HeLa. HeLa cells were transiently transfected with pCDNA6.2-WBP2 in various combinations with pCI-Neo-MST1, MST2, LATS1, LAST2, SAV, and MOB and TOPFlash luciferase reporter plasmids. Empty vector pCDNA6.2 or pCI-Neo was used as the negative control. Thirty-six hours after transfection, the cells were stimulated with L control-(O) and WNT3A-conditioned medium for 8h, followed by dual luciferase assay. **b** QC of the Hippo components (MST1, MST2, LATS1, LATS2, SAV, MOB) overexpression and their activation in HeLa using immunoblotting. **c**: Overexpression of Hippo pathway cassettes downregulated WBP2 protein expression in a dose-dependent manner in HeLa. HeLa cells were transiently transfected with pCDNA6.2-WBP2 in various combinations with pCI-Neo-MST1, MST2, LATS1, LAST2, SAV and MOB in the decreasing dose. Twenty-four hours after transfection, the total cell lysates were examined by western blot. **d** (i) LATS1/2 knockdown did not abolish MST1/2-mediated WBP2 downregulation in HeLa. HeLa cells were transiently transfected with pCDNA6.2-WBP2 together with vector or pCI-Neo-MST1, MST2, and SAV in the presence luciferase or LATS1+2 siRNAs. Twenty-four hours after transfection, the total cell lysates were examined by western blot. (ii) LATS1/2-mediated WBP2 downregulation was independent of MST1/2 knockdown in HeLa. HeLa cells were transiently transfected with pCDNA6.2-WBP2 together with vector or pCI-Neo-LATS1, LATS2 and MOB in the presence luciferase or MST1+2 siRNAs. Twenty-four hours after transfection, the total cell lysates were examined by western blot. **e** SAV potentiates MST1 and/or MST2-mediated downregulation of WBP2 in HeLa. HeLa cells were transiently transfected with pCDNA6.2-WBP2 together with MST1, MST2 and SAV individually and in combination. Twenty-four hours after transfection, the total cell lysates were examined by western blot.

To test the effect of Hippo signaling on WBP2, we screened MST, LATS, SAV, and MOB in different combinations on WBP2-mediated Wnt activity^15,16^. All the combinations involving MST resulted in a more significant abolishment of WNT3A-induced, WBP2-mediated TCF reporter activity than that with LATS (Fig. 1a). Consistently, MST also significantly reduced WBP2/WNT3A-induced AXIN2 mRNA and/or protein expression (Fig. S1A). The anticipated effects of the various cassettes on downstream YAP were observed (Fig. 1b).

WBP2 is regulated mainly via protein stability^15^. Hence, we tested if Hippo pathway inhibited WBP2 function by downregulating its expression. Figure 1c shows that MST and/or LATS cassettes led to a significant dose-dependent loss of WBP2 protein expression. Both MST and LATS kinases downregulated WBP2, although MST was more potent. To address whether they act in the same signaling axis, the effect of LATS knockdown on MST-mediated WBP2 downregulation and vice versa were examined. Figure 1d, i demonstrated that the action of MST1/2 on WBP2 was unaffected when LATS1/2 was knocked down (Fig. S1B, i). Similarly, MST1/2 knockdown did not rescue the LATS1/2-mediated WBP2 downregulation (Fig. 1d, ii), as LATS1/2 overexpression in the absence/presence of MST1/2 knockdown both led to a similar 60% decrease in WBP2 level (Fig. S1B, ii). This suggests that MST and LATS operate independently and/or in parallel to downregulate WBP2 in a non-canonical fashion.

Since MST is more potent on WBP2 compared to LATS, subsequent studies focused on MST. Further analysis showed that SAV alone did not affect WBP2 expression but its presence augmented MST1/2-mediated WBP2 degradation (Fig. 1e). MST1 or MST2 alone downregulated WBP2 expression, albeit MST1’s effect was stronger than MST2.

### MST inhibition of WBP2 is kinase-dependent and enhanced by SAV

**Fig. 2:**
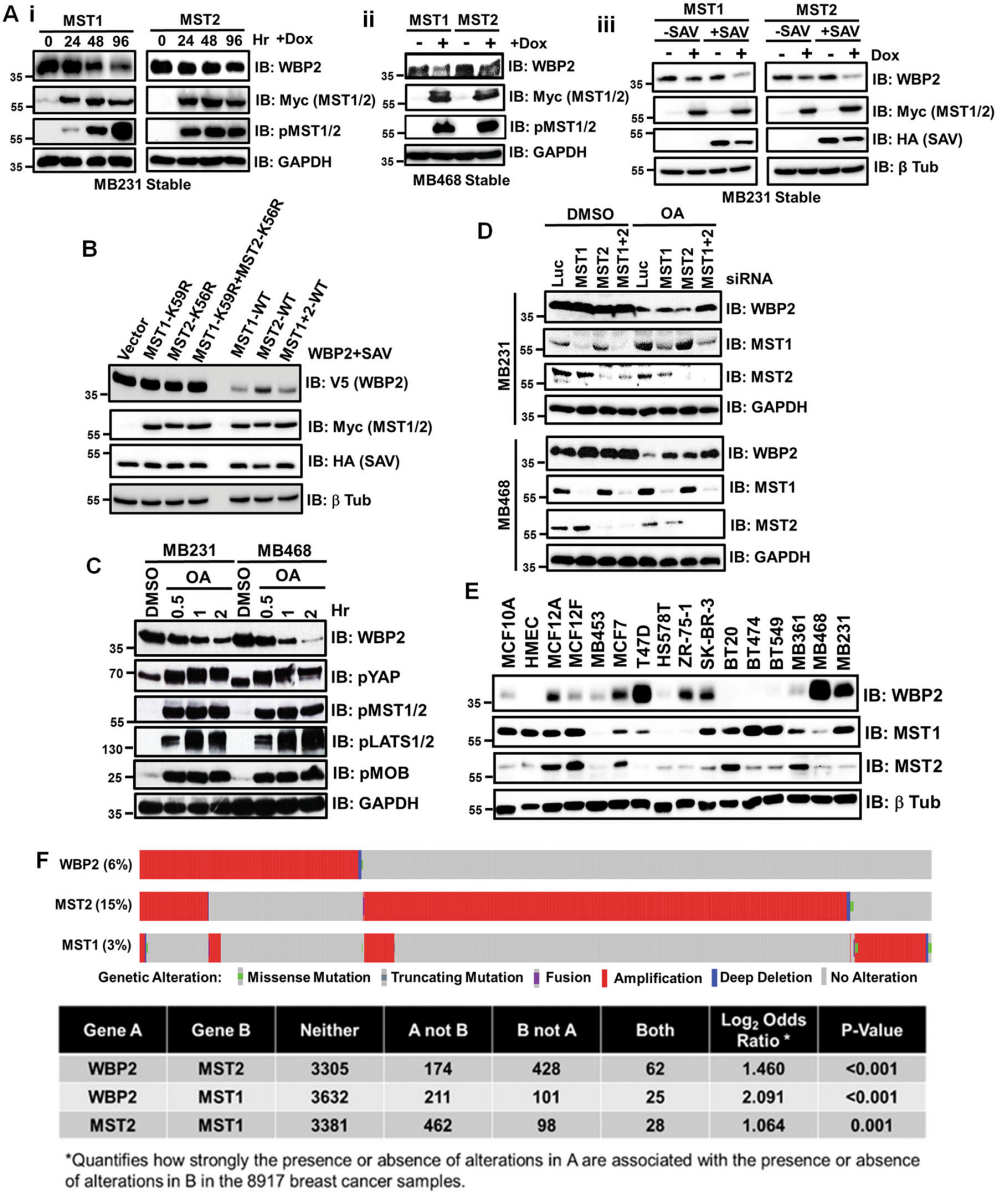
MST Inhibition of WBP2 is Kinase-dependent and Enhanced by SAV. **a** MST1 or MST2 overexpression downregulates endogenous WBP2 protein level in the temporal-dependent manner in MDA-MB-231 and MDA-MB468. MDA-MB-231 (i) or MDA-MB-468 T-Rex cells (ii) stably infected with MST1 or MST2 was induced with doxycycline for various time courses (0–72h). The total cell lysates were examined by western blot. (iii) SAV potentiated MST1/2-mediated downregulation of WBP2 in MDA-MB-231. Stable MDA-MB-231 T-Rex cells stably infected with MST1 or MST2 was transiently transfected with vector or pCI-Neo-SAV, followed by 48h doxycycline induction. The total cell lysates were then examined by western blot. **b** MST1/2 downregulates WBP2 protein expression in the kinase activity-dependent manner in HeLa. HeLa cells were transiently transfected with pCDNA6.2-WBP2 and pCI-Neo-SAV together with vector, wild-type (WT) or kinase-dead (MST1-K59R, MST2-K56R) pCI-Neo-MST1 and/or MST2 kinases downregulated WBP2 expression in HeLa. Twenty-four hours after transfection, the total cell lysates were examined by western blot. **c** Activation of MST1/2 and LATS1/2 kinases via okadaic acid (OA) induced temporal-dependent WBP2 downregulation in MDA-MB-231 and MDA-MB-468. MDA-MB-231 and MDA-MB-468 cells were treated with vehicle control (DMSO) or OA for various time courses (0–2h). The total cell lysates were then examined by western blot. **d** MST1/2 knockdown significantly abolished OA-induced WBP2 downregulation in MDA-MB-231 and MDA-MB-468. MDA-MB-231 and MDA-MB-468 cells were transiently transfected with luciferase or MST1+2 siRNAs. Forty-eight hours after transfection, the cells were treated with vehicle control (DMSO) or OA for 2h. The total cell lysates were then examined by western blot. **e** Immunoblot analysis of WBP2 and MST1 or MST2 protein expression in vitro in a panel of breast cancer cell lines. **f** In silico association analysis between the genomic alterations of WBP2, MST1 or MST2, in clinical breast cancers. The map demonstrates the inverse alteration pattern of WBP2 and MST1/2 genes in individual cancer patients. The figure was generated based on the patients’ genomic data analysis by cBioPortal tool obtained from 8917 heterogeneous patients selected from 10 studies including Breast Cancer (METABRIC, Nature 2012 & Nat Commun 2016), Breast Invasive Carcinoma (Broad, Nature 2012), Breast Invasive Carcinoma (Sanger, Nature 2012), Breast Invasive Carcinoma (TCGA, Cell 2015), Breast Invasive Carcinoma (TCGA, Firehose Legacy), Breast Invasive Carcinoma (TCGA, Nature 2012), Breast Invasive Carcinoma (TCGA, PanCancer Atlas), Metastatic Breast Cancer (INSERM, PLoS Med 2016) and The Metastatic Breast Cancer Project (Provisional, February 2020). The continuous bar is made up of individual columns representing the status of gene alteration in individual patients. % refers to frequency of gene alteration in the studied population of breast cancer clinical samples.

Triple-negative breast cancer (TNBC) cell lines highly express WBP2^15^. To examine if MST regulates WBP2 in TNBC cells, stable inducible MST1 and MST2 system was generated from MDA-MB-231 and MDA-MB-468 cells. Figure 2a, i and ii showed that endogenous WBP2 was decreased upon doxycycline-induced MST1/2 expression. Exogenous SAV expression further potentiated the effect (Fig. 2a, iii). Non-specific effect of Doxycycline on WBP2 was ruled out as Doxycycline had no effect on endogenous WBP2 in vector control cells (Fig. S1C).

The degree of WBP2 downregulation seems to correlate with the time-dependent phosphorylation of MST1/2 following doxycycline treatment (Fig. 2a, i). Non-specific activation of pMST1/2 by doxycycline was ruled out as the phosphorylation of MST1/2 in MST1/2-transfected MDA-MB231 cells was detected regardless of the presence/absence of doxycycline (Fig. S1D). This suggest that overexpression may lead to auto-phosphorylation/activation of MST1/2. MST1/2 were functionally active as indicated by the increased phosphorylated MOB (Fig. S1F). Figure 2b shows that kinase-dead mutants of MST1-K59R and MST2-K56R, which are defective in phosphorylating downstream MOB and LATS kinases^17^, failed to downregulate WBP2 expression. Thus, kinase activity is critical for the MST regulation of WBP2.

Okadaic acid (OA) is commonly used to activate Hippo pathway by enhancing phosphorylation/activation of MST1/2^18–20^. Figure 2c shows that OA treatment in MDA-MB-231 and -468 cells resulted in a time-dependent downregulation of WBP2, concomitant with activation of Hippo kinases (MST, LATS), regulators (MOB) and effector (YAP). MST was proven to mediate the OA-induced WBP2 downregulation as double knockdown of MST1/2 in MDA-MB-231 and MDA-MB-468 cells rescued OA-induced WBP2 downregulation (Fig. 2d).

Next, we analyzed the protein expression of MST1, MST2, and WBP2 in a panel of breast cancer cell lines to determine the correlation between MST and WBP2 expression (Fig. 2e). Although not statistically significant, Fig. S1E shows an inverse relationship between MST1/2 and WBP2 protein expression. Significant associations between lower MST1 or MST2 with higher WBP2 expression were observed in clinical samples (Fig. 2f) when the mRNA expression profiles of MST1/2 and WBP2 in the TCGA RNA-seq database were analyzed using cBioPortal^21,22^.

### MST inhibits WBP2-driven breast cancer resulting in good prognosis in xenografts

**Fig. 3:**
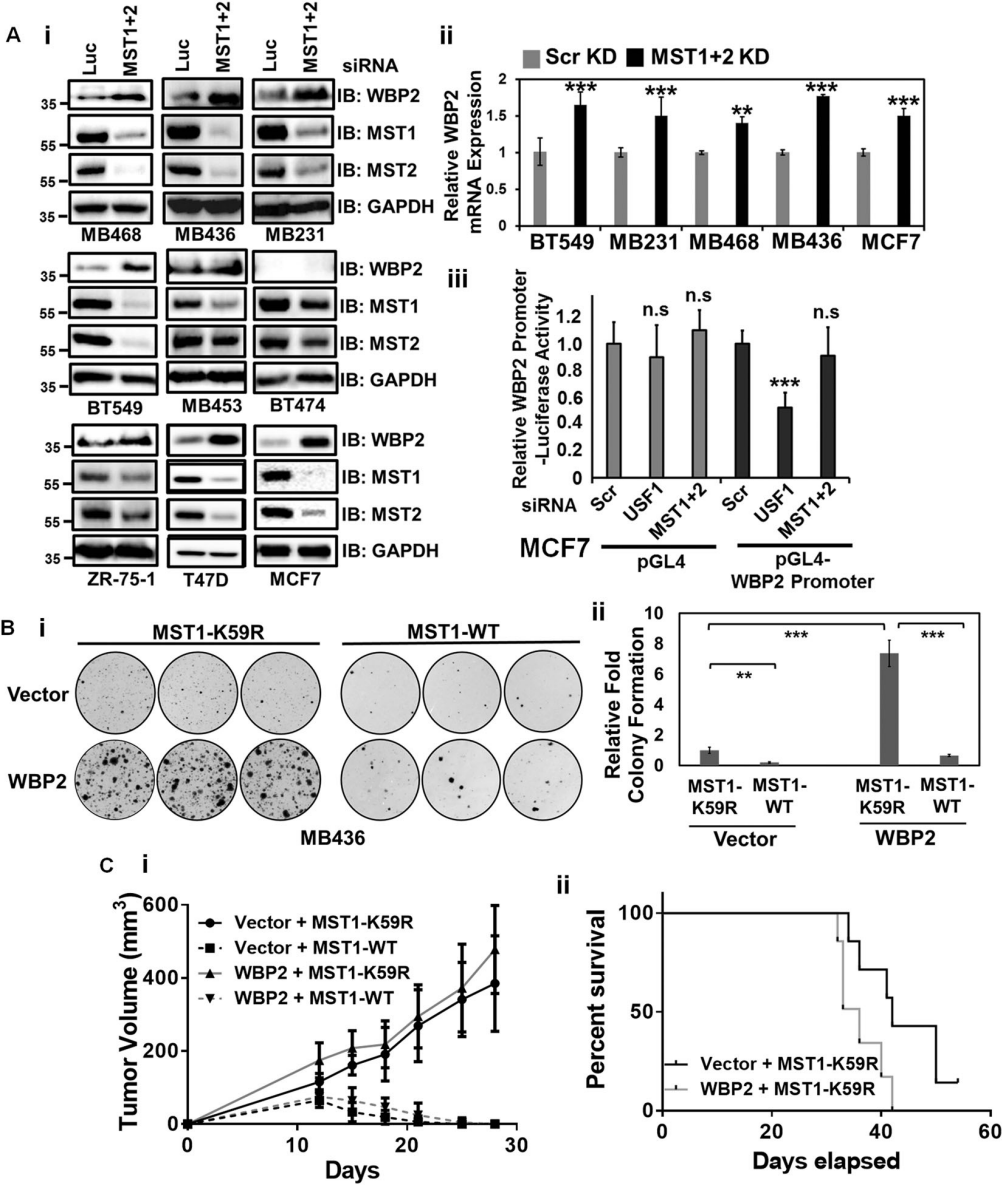
MST Inhibits WBP2-driven Breast Cancer Resulting in Good Prognosis in Xenografts. **a** MST1&2 negatively regulates WBP2 expression in multiple breast cancer cells. A panel of breast cancer cell lines were transiently transfected with luciferase or MST1+2 siRNAs. Forty-eight hours after transfection, WBP2 protein (i) and mRNA (ii) expression was examined by western blot and qPCR. (iii) MST1/2 USF-1 knockdown but not MST1/2 knockdown in MCF7 decreased the WBP2 promoter luciferase reporter activity. **b** MST1-WT but not kinase-dead K59R mutant significantly abolished the WBP2-driven colony growth of MDA-MB-436 cells. Stable vector- and WBP2-WT-overexpressing MDA-MB-436 cells were transiently transfected with pCI-Neo-SAV and MST1-K59R or MST1-WT for 24h before being seeded for in vitro clonogenic assay and monitored up to 14 days in the presence of hygromycin. (i) Scanned image showing different levels of colony formation in these transfected cells. (ii) Quantitative analysis of the colony formation using the ImageJ software. **c** MST1-WT but not kinase-dead K59R mutant significantly abolished the WBP2-driven tumor xenograft growth of MDA-MB-436 cells. Stable vector- and WBP2-WT-overexpressing MDA-MB-436 cells were transiently transfected with pCI-Neo-SAV and MST1-K59R or MST1-WT for 24h before being harvested for tumor xenograft study. i The time course line plot of in vivo growth of xenografted tumors. ii Kaplan–Meier survival plot of the xenografted tumor-bearing mice from MST1-K59R-transfected vector or WBP2-WT overexpressing groups. (*n* =7 mice).

To understand the MST function at the physiological level, we examined if MST acts as a rheostat to control endogenous WBP2 level via MST1&2 knockdown in a panel of breast cell lines. Increase in WBP2 protein expression was observed in 89% (8 out of 9) of the breast cancer cell lines studied (Fig. 3a, i). The results support the notion that MST is a rheostat that controls the basal level of WBP2 in a diverse molecular subtypes of breast cancer including the TNBC (MDA-MB-468, MDA-MB-436, MDA-MB-231, BT549, and MDA-MB-453).

To elucidate the mode of action of MST, we investigated if MST regulates WBP2 mRNA level. MST1&2 knockdown upregulated WBP2 mRNA (Fig. 3a, ii) but not at the transcriptional level as WBP2 promoter activity was not affected by the MST1/2 knockdown (Fig. 3a, iii). Knockdown of USF-1, the transcription factor of WBP2^23^ was used as the positive control. This suggest regulation of WBP2 by MST at the post-transcriptional/translational level.

Since WBP2 promoted in vitro and in vivo tumorigenesis^15^, we investigated if MST/SAV negates WBP2’s function in cancer. MST1-WT but not kinase-dead-K59R mutant significantly abolished the WBP2-driven colony growth of MDA-MB-436 TNBC cells (Fig. 3b, i and ii) and WBP2-mediated anchorage-independent growth of HeLa cells (Fig. S2A). Moreover, MST1-WT but not MST1-K59R blocked in vivo growth of tumor xenografts from WBP2-overexpressing MDA-MB-436 (Fig. 3c, i) and HeLa cells (Fig. S2B). WBP2-overexpressing HeLa tumor xenografts in the MST1-K59R mice group appeared to form larger tumors when compared to the vector control (WBP2 alone) group. However, the difference was not statistically significant, indicating that the bulk of the in vitro and in vivo tumor growth from WBP2-WT and MST1-K59R co-expression were contributed by WBP2. Similarly, overexpression of WBP2-WT alone resulted in more significant increase in 3D growth than that of MST1-K59R in MDA-MB-436 cells (Fig. S2C). This effect of WBP2 was further potentiated by co-expression of MST1-K59R. MST1-K59R mutant may confer a less dominant gain-of-function activity but the investigation is outside the scope of this study.

All the WBP2-overexpressing MDA-MB-436 tumor xenografts in the MST1-WT mice group completely regressed by 28 days. Hence, this was taken as the endpoint of the study. Comparing the in vivo growth of vector vs WBP2-overexpressing MDA-MB-436 tumor xenografts in the MST1-K59R mice group showed that tumor xenografts with WBP2 overexpression generated bigger tumor volume in overall. To further determine if this bigger tumor volume correlates to poorer prognosis, we continued to monitor the survival of these 2 groups up to 55 days to allow any difference in survival to show up. Breast cancer cells co-expressing MST1-K59R and WBP2 significantly decreased the overall survival of the animals compared to those expressing MST1-K59R alone (Fig. 3c, ii) supporting WBP2 and MST as prognostic factors.

### Dicer mediates the negative effect of MST on WBP2 expression and function

Since ITCH is a negative regulator of WBP2 protein^15^, we investigated if ITCH mediates the MST-induced WBP2 downregulation. ITCH knockdown did not significantly rescue the MST-SAV- and OA-induced WBP2 downregulation in HeLa (Fig. S3A, i), MDA-MB-231 and MDA-MB-468 (Fig. S3A, ii). Proteasomal/lysosomal degradation was also ruled out as proteasome (MG132, Lactacystin) and lysosomal (Chloroquine, Concanamycin A) inhibitors failed to block MST-SAV (Fig. S3B, i) or OA-induced WBP2 degradation (Fig. S3B, ii). It is possible that the binding of TAZ/YAP protects WBP2 from MST1. Fig. S3C showed that TAZ-WT or YAP-WT overexpression only moderately rescued MST1-induced WBP2 downregulation, suggesting the presence of a yet unknown mediator in the MST-WBP2 axis.

**Fig. 4:**
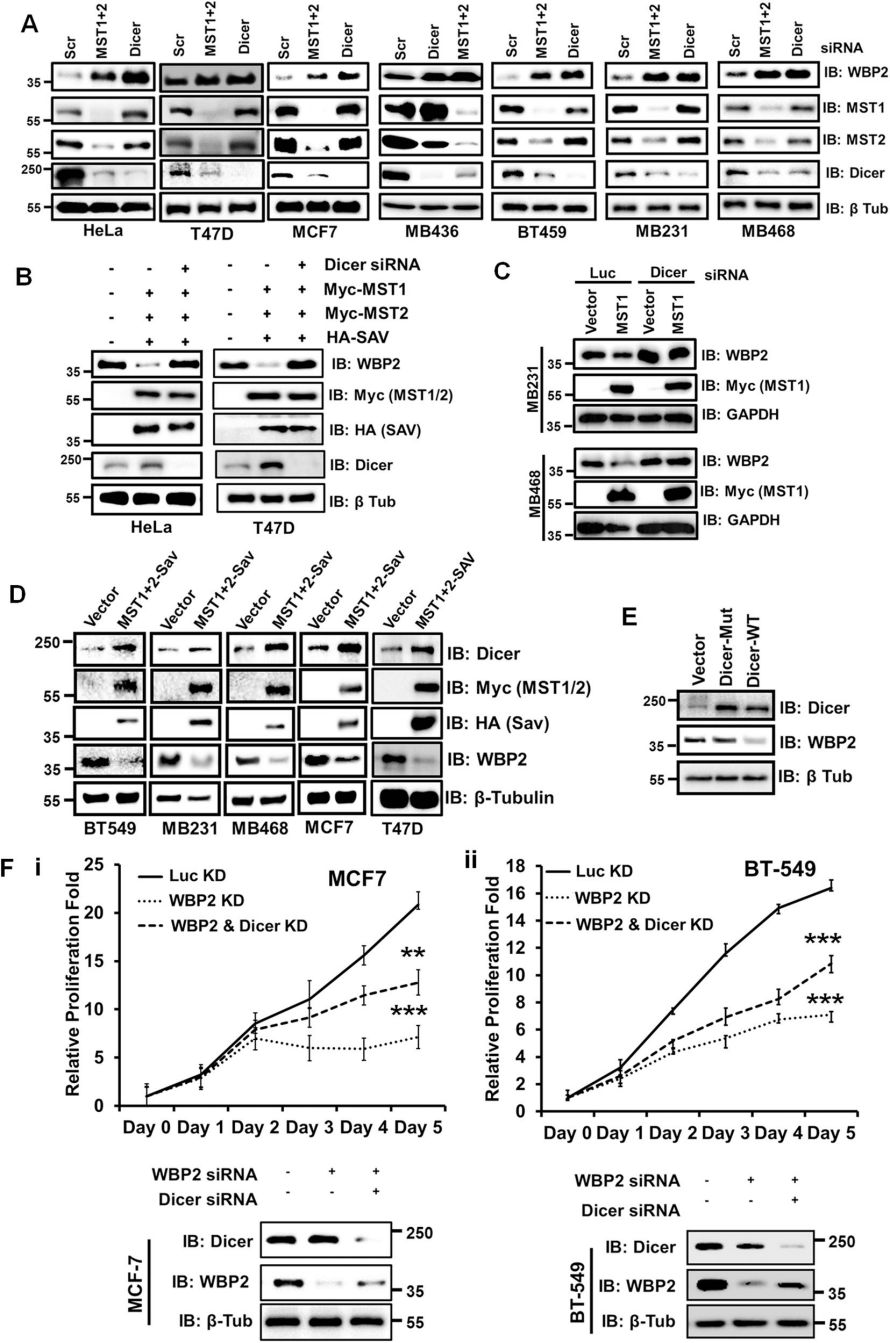
Dicer Mediates the Negative Effect of MST on WBP2 Expression and Function. **a** MST1/2 or Dicer knockdown upregulates endogenous WBP2 protein level in multiple breast cancer cells. A panel of breast cancer cell lines were transiently transfected with MST1&2 or Dicer siRNAs. Seventy-two hours post-transfection, the total lysates were examined by western blot. **b** Dicer knockdown rescues MST1/2-SAV-mediated WBP2 downregulation in HeLa and T47D. HeLa and T47D cells were transiently transfected with pCDNA6.2-WBP2 and vector or pCI-Neo-MST1, MST2 & SAV in the presence of luciferase or Dicer siRNAs. Seventy-two hours after transfection, total cell lysates are examined by western blot. **c** Dicer knockdown rescues MST1-induced WBP2 downregulation in MDA-MB-231 and MDA-MB-468. MDA-MB-231 and MDA-MB-468 T-Rex stable vector- or MST1-expressing cells were transiently transfected with luciferase or Dicer siRNAs and induced with doxycycline for 48h. The total lysates are examined by western blot. **d** MST1&2-SAV overexpression downregulates endogenous WBP2 protein level in multiple breast cancer cells. A panel of breast cancer cell lines were transiently transfected with vector or pCI-Neo-MST1, MST2 and SAV plasmids. Seventy-two hours post-transfection, the total lysates were examined by western blot. **e** Dicer specifically regulated WBP2 protein through its miRNA processing activity in BT549. BT549 cells were transiently transfected with vector, Dicer-Mut with in-frame deletion of its catalytic RIIID domain and Dicer-WT. Forty-eight hours after transfection, the total lysates were examined by western blot. **f** Dicer knockdown partially rescued the WBP2 knockdown-induced decreased proliferation of MCF7 (i) or BT549 (ii) cells via partial restoration of WBP2 expression. MCF7 or BT549 cells were transiently transfected with luciferase, WBP2 and/or Dicer siRNAs for 48h before being seeded for quantitative measurement of cell proliferation over 5 days via MTS assay. Total lysates were examined by western blot.

Next, we assessed the role of miRNAs as a major player in the regulation of WBP2 by MST. Figure 4a shows that knockdown of Dicer, a key miRNA processor, in multiple cell lines led to robust upregulation of endogenous WBP2 protein, phenocopying MST1/2 knockdown. The off-target effect of siRNA is unlikely since another set of siRNAs for Dicer (Fig. S4A) and MST1/2 (Fig. S4B) with distinct sequences produced the same effect. MST1/2 knockdown also downregulated endogenous Dicer (but not the other way round), suggesting that MST kinase regulates WBP2 expression through Dicer-mediated miRNA biogenesis. Indeed, Dicer knockdown rescued MST1/2-mediated downregulation of WBP2 in HeLa/T47D (Fig. 4b) and MDA-MB-231/MDA-MB-468 (Fig. 4c). Reciprocally, MST1/2 overexpression led to increased Dicer with concomitant downregulation of WBP2 (Fig. 4d). DGCR8, another key miRNA processor, was also found to downregulate WBP2 in BT549 (Fig. S4C).

To further confirm that WBP2 is regulated through the miRNA-processing function of Dicer, a catalytic-mutant form of Dicer was created via an in-frame deletion in the conserved catalytic amino acids of Dicer RNaseIII domain^24^. Wild-type Dicer but not Dicer-Mut downregulated WBP2 protein (Fig. 4e), indicating that Dicer specifically regulates WBP2 through its miRNA processing activity.

Could silencing Dicer, thus increasing WBP2 expression, rescue the diminished proliferation caused by WBP2 knockdown? BT-549 TNBC and MCF7 ER + BC were suitable for this study as both have moderate expression of WBP2. MCF-7 was included to examine if the effect of Dicer on WBP2 could be reproduced in other molecular subtype besides TNBC. Dicer knockdown partially reduced the WBP2 knockdown-induced decrease in proliferation of MCF7 (Fig. 4f, i) and BT549 (Fig. 4f, ii) cells putatively due to the partial rescue of WBP2 expression.

Taken together, MST acts upstream to positively regulate Dicer which in turn downregulates WBP2 expression and function in BC.

### MST-Dicer signaling axis downregulates WBP2 via miRNA-23a

**Fig. 5:**
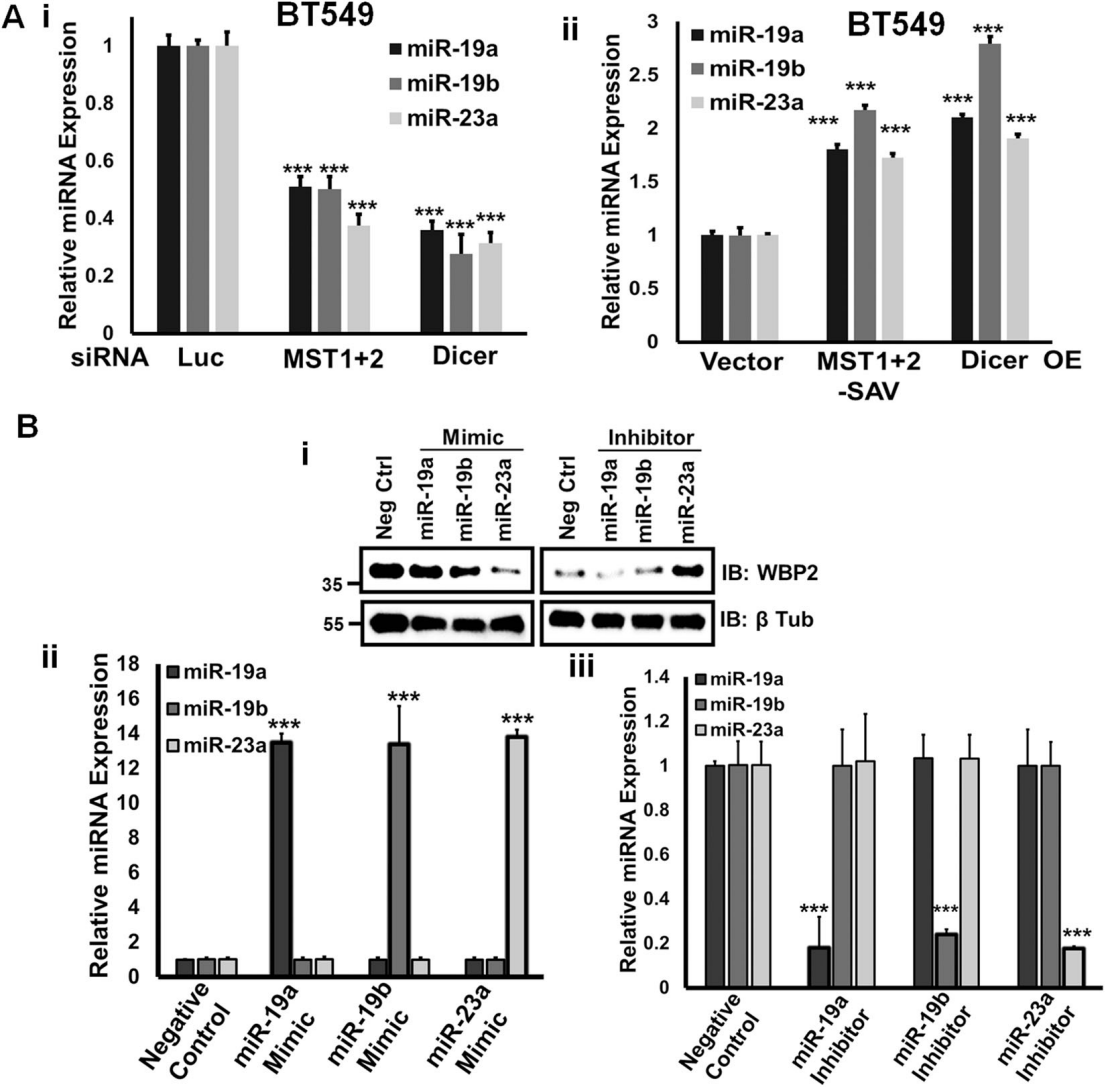
miR-23a Negatively Regulates WBP2 Protein Expression. **a** MST1/2 or Dicer knockdown (i) and overexpression (ii) downregulates and upregulates miR-19a, 19b, and 23a expression respectively in BT549. BT549 cells were transiently transfected with MST1&2 or Dicer siRNAs. Forty-eight hours after transfection, the miRNA expression for miR-19a, 19b, and 23a was examined by qPCR. **b** The miR-23a, but not miR-19a and -19b mimic and inhibitor downregulates and upregulates the endogenous WBP2 protein expression respectively in HeLa. The HeLa cells were transiently transfected with mimics and inhibitors of miR-19a, 19b, and 23a. Forty-eight hours post-transfection, the total lysates were examined by western blot (i). Expression of miR-19a, 19b, and 23a following transfection of the corresponding mimics (ii) and inhibitors (iii) were validated via qPCR.

We proceeded to identify novel WBP2-targeting miRNA(s) downstream of the MST/Dicer signaling axis. To this end, we employed 12 in-silico prediction tools to predict the miRNA(s) with a target site on WBP2 mRNA. MiR-19a, miR-19b, and miR-23a were selected for further studies due to their high prediction frequency (at least 6) across various algorithms (Table S1) and their relevance to Hippo pathway^25,26^. All 3 miRNAs were downregulated and upregulated upon MST1/2 or Dicer knockdown and overexpression in BT549, respectively (Fig. 5a, i and ii). This supports that miR-19a, miR-19b and miR-23a are potential WBP2 regulators downstream of the MST kinases-Dicer regulatory network.

To validate WBP2 as an authentic target of miR-19a/19b/23a, miR-mimics and inhibitors were used. Only miR-23a mimic and inhibitor successfully downregulated and upregulated the WBP2 protein expression, respectively, in HeLa cells (Fig. 5b, i) even though all 3 miRNAs were properly manipulated through the mimic/inhibitors (Fig. 5b, ii and iii). This positions miR-23a as the most robust regulator of WBP2. Indeed, the Dicer-induced miR-23a expression could be abolished by the mutation in its catalytic domain^24^ (Fig. S4D).

### miRNA-23a directly targets WBP2 3′UTR and their expression levels are inversely correlated in clinical breast cancer samples

**Fig. 6:**
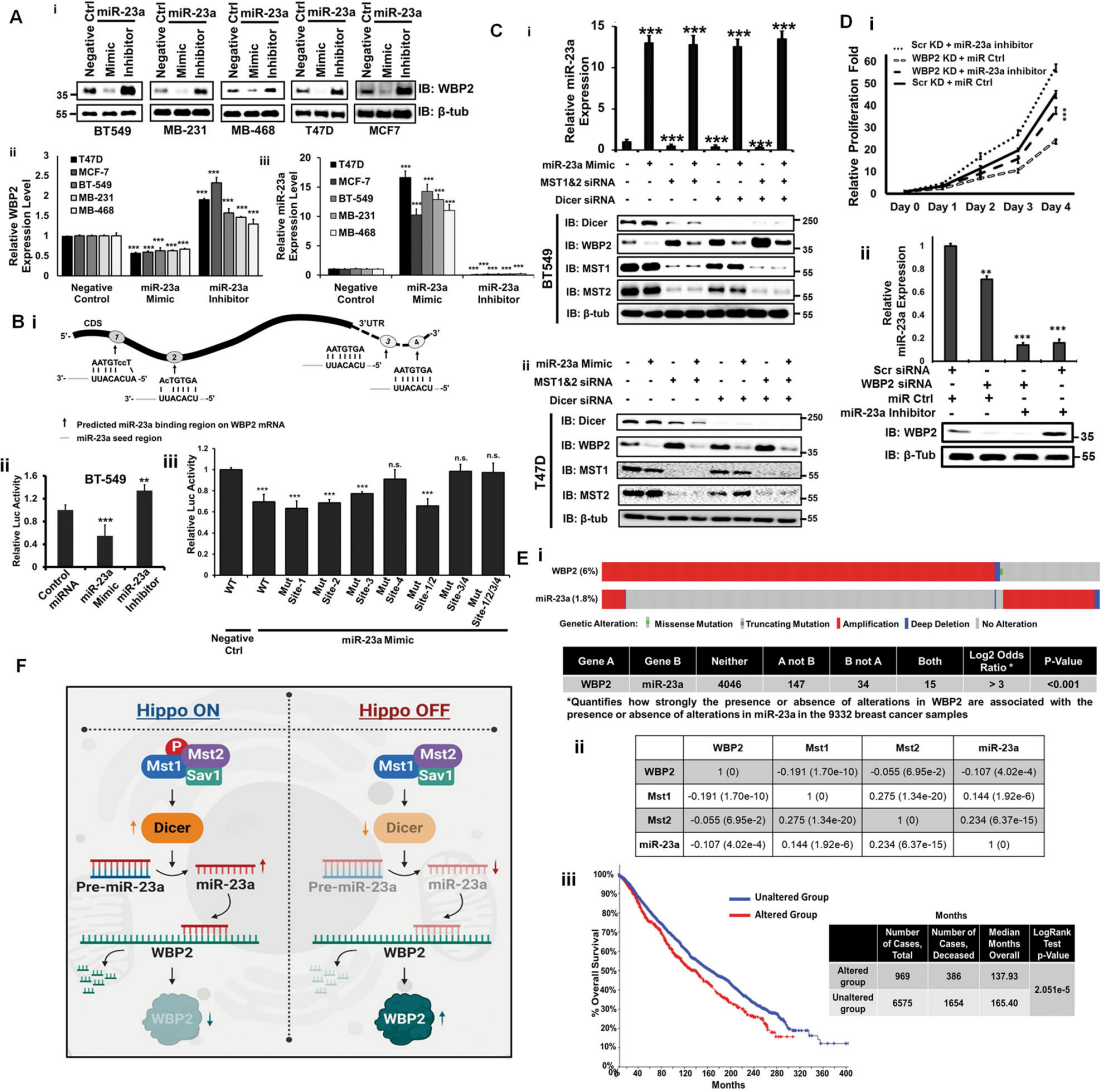
miRNA-23a Directly Targets WBP2 3′UTR and Their Expression Levels are Inversely Correlated in Clinical Breast Cancer Patients. **a** The miR-23a mimic and inhibitor downregulates and upregulates the endogenous WBP2 protein expression respectively in multiple breast cancer cells. The MCF7, T47D, BT549, MDA-MB-231, and MDA-MB-468 cells were transiently transfected with mimics and inhibitors of miR-23a. Forty-eight hours post-transfection, endogenous WBP2 protein (i) and mRNA (ii) expression as well as QC of miR-23a expression (iii) was examined via western blot and qPCR. **b** (i) Potential target sites of miR-23a in the CDS and 3′-UTR regions of WBP2 mRNA. (ii) BT-549 cells were transfected with psiCheck2-WBP2 mRNA (CDS+3′UTR) in the presence of miR-23a mimic and inhibitor. After 24h, cells were lysed and renilla/firefly luciferase activity was measured. (iii) Mapping of the binding site for the miR-23a-WBP2 interaction using the WT and various mutants of Site-1 to 4 via renilla/firefly dual luciferase assay. **c** Overexpression of miR-23a abolished the MST1/2 and/or Dicer siRNA knockdown-induced upregulation of WBP2 protein expression in (i) BT549 and (ii) T47D. BT549 and T47D cells were transiently transfected with luciferase, MST1&2 and/or Dicer siRNAs in the presence of miRNA control or miR-23a mimic. Forty-eight hours post-transfection, the total lysates and QC of miR-23a expression were examined by western blot and qPCR respectively. **d** (i) WBP2 siRNA knockdown rescued the miR-23a inhibitor-induced cell proliferation of BT549. BT549 cells were transiently transfected with scramble or WBP2 siRNAs and miR control or inhibitor for 48h before being seeded for quantitative measurement of cell proliferation over 4 days via MTS assay (ii) miR-23a and WBP2 expression were verified by qPCR and western blot, respectively. **e** (i) In silico association analysis between the genomic alterations of WBP2 and miR-23a in clinical breast cancers. The map demonstrates the inverse alteration pattern of WBP2 and miR-23a genes in individual cancer patients. The figure was generated based on the patients’ genomic data analysis by cBioPortal tool obtained from 8917 heterogeneous patients selected from 10 studies including Breast Cancer (METABRIC, Nature 2012 & Nat Commun 2016), Breast Invasive Carcinoma (Broad, Nature 2012), Breast Invasive Carcinoma (Sanger, Nature 2012), Breast Invasive Carcinoma (TCGA, Cell 2015), Breast Invasive Carcinoma (TCGA, Firehose Legacy), Breast Invasive Carcinoma (TCGA, Nature 2012), Breast Invasive Carcinoma (TCGA, PanCancer Atlas), Metastatic Breast Cancer (INSERM, PLoS Med 2016) and The Metastatic Breast Cancer Project (Provisional, February 2020). The continuous bar is made of individual columns representing the status of gene alteration in individual patients. % refers to frequency of gene alteration in the studied population of breast cancer clinical samples. (ii) Correlational analysis among the mRNA levels of MST1/2-miR-23-WBP2 signature components in breast invasive carcinoma cases obtained from ENCORI database. Data are shown as *r* (*p*-value), Pearson correlation test. (iii) Survival analysis of MST1/2, miR-23a, and WBP2 genes using cBioPortal database. **f** A schematic representation of the pathway showing MST-Dicer-miR-23a mediated WBP2 downregulation.

In addition to HeLa, Fig. 6a shows that miR-23a mimic downregulated WBP2 protein (Fig. 6a, i) and mRNA (Fig. 6a, ii) expression in MCF7, T47D, BT549, MDA-MB-231, and MDA-MB-468. Consistently, miR-23a inhibitor upregulated endogenous WBP2 protein (Fig. 6a, i) and mRNA (Fig. 6a, ii) expression, supporting the notion that miR-23a controls the basal state of WBP2 in breast cancer cells. The effect of miR-23a on WBP2 protein level appears to be more pronounced than WBP2 mRNA level.

Next, we used the TargetScan^27^ and RNA22 (https://cm.jefferson.edu/rna22/Interactive/) tools to predict the binding site(s) of miR-23a on WBP2 mRNA. Four putative WBP2 sequences were found, two at the 3′UTR with perfect complementarity and two at the CDS with partial complementarity to miR-23a (Fig. 6b, i).

To investigate whether miR-23a directly targets WBP2 by binding to its mRNA (CDS + 3′UTR) and to map the binding site(s), WT or mutant target sequences were co-transfected with miR-23a mimic or inhibitor into BT549 and luciferase assay performed. The miR-23a mimic inhibited the luciferase activity of psiCheck2-WBP2-WT, while the inhibitor significantly promoted the reporter activity (Fig. 6b, ii). Only mutation of the WBP2 3′UTR (psiCheck2-WBP2-Mut-Site4 but not -Mut-Site1, 2, or 3) ablated the inhibitory effect of miR-23a on luciferase (Fig. 6b, iii). Hence, miR-23a binds specifically and directly to Site4 within the 3′UTR of WBP2.

The data appears to conflict with the data in Fig. 1 where MST downregulated exogenous WBP2 without the 3′UTR. This invokes the involvement of non-UTR-targeting miRNAs of WBP2. Indeed, endogenous WBP2 was downregulated to a greater extent by Dicer overexpression compared to miR23a mimics (Fig. S4E). Furthermore, miR-23a inhibitor only partially rescued the Dicer overexpression-mediated WBP2 downregulation (Fig. S4F). More than 1 miRNA, including CDS-targeting ones, could be regulating WBP2. All the reported WBP2-targeting miRNAs, i.e. miR-206^28^, miR-613^29^ or miR-485^30^, target WBP2 3′UTR.

To confirm that exogenous WBP2 could also be regulated by miRNA(s), Dicer was co-overexpressed with V5-tagged WBP2. The non-specific effect of Dicer-induced miRNAs on the V5 sequence was ruled out since Dicer overexpression only downregulated the V5-WBP2 and not V5-p47 and V5-NRD controls (Fig. S4G). The results confirmed that the WBP2 CDS can be targeted by Dicer-induced miRNAs. A list of high-scoring WBP2 CDS-targeting miRNAs is shown in Table S2. The ones in BOLD were selected for further validation based on their expression pattern (lower) in breast tumors vs normal tissues. However, overexpression of their mimics did not affect the exogenous WBP2 (Fig. S4H). Other candidate WBP2 CDS-targeting miRNAs remain to be tested as potential mediators of MST.

To elucidate the role of miR-23a in MST/Dicer-mediated downregulation of WBP2 expression, we studied the combinatorial effects of MST1/2, Dicer, and miR-23a mimic on WBP2 expression. MST1/2 and/or Dicer knockdown all led to the upregulation of WBP2 expression owing to downregulation of miR-23a since this phenotype was abolished by miR-23a mimic (Fig. 6c). This implies a MST→Dicer→miR-23a→WBP2 signaling axis in both BT549 and T47D. Indeed, overexpression of miR-23a in BT549 cells increased cell proliferation that could be rescued by WBP2 knockdown (Fig. 6d).

The above in vitro observations raised a testable hypothesis that the expression of miR-23a and WBP2 are inversely correlated in the clinical setting. Hence, the expression profiles of WBP2 and miR-23a in breast cancer samples were analyzed using cBioPortal database. The map demonstrated an inverse relationship between WBP2 and miR-23a alterations (Fig. 6e, i), thereby supporting their clinical relevance.

To further validate the inverse correlation between WBP2 and miR-23a, ENCORI database was employed. Unlike the cBioPortal, which analyzes the association between genomic alterations qualitatively, this database uses the quantitative expression profiles of genes obtained from the TCGA RNA-seq data. Consistent with the cBioPortal database, the negative correlation between WBP2 and miR-23a was observed in breast invasive carcinoma clinical samples. Moreover, TCGA meta-analysis showed a downregulation of miR-23a in breast invasive carcinoma samples, reflecting that WBP2 oncogene is regulated by a potential tumor suppressor miR-23a in breast cancer (Fig. S4I).

Next, we incorporated MST1/2 to the in silico analysis. A significant positive correlation between MST1/2 and miR-23a was observed. Interestingly, the expression profile of these two kinases MST/12 inversely correlated with WBP2 expression level (Fig. 6e, ii). This suggests that higher WBP2 expression in clinical BC could result from suppressed levels of its upstream negative regulators like MST1/2 and miR-23a.

Given the negative correlation of MST1/2-miR-23a with WBP2, and positive correlation between MST1-MST2, MST1-miR-23a, and MST2-miR-23a, we questioned whether this gene signature could serve as predictive biomarkers. Survival analysis was performed using the cBioportal database as it allows survival analysis of a panel of genes. The results showed that co-alterations of the MST1/2-miR-23a-WBP2 corelated with poorer survival in breast cancer patients (Fig. 6e, iii). Collectively, the in vitro and in silico data support the notion that the MST1/2 → miR-23a → WBP2 could have a clinical significance in breast cancer.

## Discussion

The question of whether WBP2 is regulated by the Hippo pathway is a pertinent one and fills a significant knowledge gap. Despite the well-known association of WBP2 with YAP and TAZ, which are negatively targeted by the Hippo/MST pathway, it remains unclear if WBP2 is similarly regulated. Here, we report that the Hippo pathway negatively regulates WBP2 expression via a novel mechanism involving Dicer. The antagonistic effect of MST and Dicer on WBP2 was demonstrated in the majority of the breast cancer cell lines tested including the TNBC and validated in xenograft models, supporting the notion that MST acts as yet another rheostat that regulates the expression of WBP2. MST/Hippo is required for the expression of Dicer, which is needed to generate WBP2-targeting such as miR-23a and possibly others yet to be identified, leading to the downregulation of WBP2 expression and oncogenic function as shown by the schematic representation in Fig. 6f. The findings provide another layer of insights into the molecular etiology of breast cancer.

There is precedence of WBP2 being a kinase substrate^16^. That kinase-dead MST mutant could not downregulate WBP2 raises a possibility that MST1/2 phosphorylates and destabilizes WBP2. However, several single Ser/Thr mutants of WBP2 created based on the predicted Ser/Thr phosphorylation sites failed to rescue MST-targeted WBP2 downregulation (Fig. S4J). It is conceivable that MST phosphorylates other substrates involved in miRNA regulation as reported in other studies on ROCK^31^, ATM kinase^32^, EGFR, and MET receptor tyrosine kinase^33^, which demonstrated the critical role of kinase activity in the regulation of miRNA biogenesis.

In the canonical Hippo signaling, LATS acts downstream of MST^34^. In this study, LATS was less potent than MST in downregulating WBP2 expression. Furthermore, our data indicate that MST and LATS kinases acted independently on WBP2. It appears that the Hippo pathway ramifies into divergent signaling cascades that exert differential control over downstream proteins e.g. WBP2 primarily by MST and YAP/TAZ mainly via LATS. LATS-independent MST-mediated or MST-independent LATS functions have been demonstrated in multiple studies. For example, the functional role of MST1 in pancreatic β cells apoptosis is LATS1/2-independent^35^. Similarly, LATS1/2 was not involved in MST1-mediated PRDX1 phosphorylation and inactivation in hydrogen peroxide-treated cells^36^.

The regulation of Dicer expression by MST reveals yet another novel mechanism through which Hippo pathway regulates miRNA biogenesis. Nuclear (active) YAP and TAZ have been shown to positively regulate Dicer expression post-transcriptionally via Let-7 miRNA^25^. High cell density-mediated sequestration of TAZ/YAP in the cytoplasm (presumably due to Hippo pathway activation), results in reduced Dicer expression and the subsequent defective processing of pre-miRNAs. This is contradictory to our data where MST positively regulates the Dicer expression. It is not clear if both regulatory axes act independently or in the feedback manner to regulate the miRNA biogenesis. Nevertheless, MST1/2-mediated regulation of Dicer expression may help to control WBP2 expression in the cytoplasm and prevent excessive WBP2 build up that might otherwise lead to increased transcriptional activity in the nucleus.

The novel MST/Dicer axis was demonstrated to downregulate WBP2 via miR-23a, which is validated to bind directly to the 3′UTR sequence of WBP2. The perfect complementary sequence of miR23a to the 3′UTR of WBP2 suggests that target mRNA degradation instead of translation inhibition is a more probable mechanism of action^37^. Our study positions miR-23a as a potential tumor suppressor by virtue of its negative effect on WBP2 expression. However, miR23a may display oncogenic role in different biological contexts^38–42^. In general, miR-23a exerts tumor-suppressive function, through the suppression of cell proliferation and induction of apoptosis via its association with p53^27,43–45^ and/or cellular senescence^46^.

Global repression of miRNA expression is a frequent phenomenon in multiple different types of cancers^47^. Furthermore, the downregulation of miRNA processing via depletion of Microprocessor components or the DICER complex leads to cellular transformation and tumorigenesis^48^. All these indicate a tumor-suppressive role for the majority of miRNAs. Our study suggests that other WBP2-targeting miRNA(s) cooperate with miR-23a to block WBP2 expression. For example, three other miRNAs that were recently reported to inhibit WBP2 expression-miR-206^28^, miR-613^29^, and miR-485^30^ were also found in our in silico analysis although they ranked lower than miR-23a, which was predicted by 7 out of the 12 algorithms used compared to 6/12 for miR-206 and 5/12 for miR-613 and miR-485 (Table S3). However, it is not clear if miR-206, miR-613, and miR-485 are targets of the MST/Dicer axis.

In conclusion, the Hippo/MST pathway is established to modulate breast cancer by controlling the expression of WBP2 via a novel mechanism involving Dicer. The study offers new insights into the regulation of the WBP2 oncogene by the MST tumor suppressor. The findings have implications in the clinical management of breast cancer.

## Materials and methods

### Antibodies and reagents

WBP2 monoclonal antibody (MABS441-clone 4C8H10) was purchased from EMD Millipore (Billerica, MA, USA). Other antibodies, plasmids, reporters, siRNA, shRNA, and miRNA sequences used are detailed in Supplementary info.

### Cell culture, ligand/inhibitor treatments, cell lysis, and immunoblotting

Cell lines used in experiments and their culture conditions as well as transfection/lentiviral transduction protocols are described in detail in Supplementary info. Doxycycline, okadaic acid, MG132, Lactacystin, concanamycin A and chloroquine were from Sigma. Cell lysis and western blot analyses were done as described in Supplementary info.

### Dual luciferase reporter assay and qPCR

The Dual-luciferase reporter assay (Promega) was performed according to the manufacturer’s instructions and quantified using Luminoskan Ascent Microplate Luminometer (Thermo-Scientific). Firefly signals were normalized to Renilla signals. RNA extraction, qPCR of mRNA and miRNA are described in Supplementary info.

### In vitro cell-based assays

The cell proliferation was measured using the CellTiter 96® Aqueous One Solution Non-Radioactive (MTS) Cell Proliferation Assay Reagent (Promega), according to the manufacturer’s instructions. The anchorage-independent growth via soft agar assay was assayed by protocol as described^49^. For clonogenic/colony formation assay, 5000 cells per well were plated in 6-well plates and allowed to grow for about 7–10 days until colonies could be clearly seen. Plates containing colonies were gently washed with PBS and fixed with 3.7% formaldehyde for 10 min. Colonies were stained with 0.2% crystal violet solution in 10% methanol for 10 min, followed by repeated washing with PBS to remove excess stain. The colony formation was quantified using the ImageJ-ColonyArea plugin^50^.

### Xenograft studies

All animal housing and handling procedures were approved and performed in accordance with the IACUC guidelines in the National University of Singapore. For xenograft tumor formation, the 5-week-old female Athymic Nude mice (In Vivos, Singapore) mice were randomly divided into three (HeLa) or four (MDA-MB-436) groups (*n* = 7/group) and inoculated subcutaneously in flanks with HeLa-WBP2 (2 × 10^6^ in 200 μl of DPBS and Matrigel 1:1; Vector, MST1-K59R & MST1-WT) or into a mammary fat pad with MDA-MB-436 (5 × 10^6^ in 200 μl of DPBS and Matrigel 1:1; Vector + MST1-K59R, Vector + MST1-WT, WBP2 + MST1-K59R, and WBP2 + MST1-WT). Tumor development was monitored and tumor volumes were measured in a blinded fashion and calculated as (width^2^ × length)/2. For survival evaluation, mice were euthanized when the tumor reached 15 mm in diameter and the day of euthanization was considered as the day of death (endpoint). The tumor volume data represents mean ± SEM. Statistical significance was determined by Mann–Whitney test. Survival analysis was computed by the Kaplan–Meier method and statistical significance was determined by log-rank (Mantel–Cox) test.

### Correlation of genomic alteration and gene expression levels in clinical specimens

cBioPortal database was used in order to study the co-occurrence analysis of genomic-seq data^21,22^. To identify the association between the alteration pattern of the genes of interest, each pair of query genes including WBP2 and MST1 or MST2 or miR-23a were imputed. The queries were statistically analyzed by the cBioportal and the related maps were plotted for the significant associations. The clinical dataset analyzed by cBioPortal was mainly based upon the data generated by the TCGA Research Network: http://cancergenome.nih.gov/. ENCORI database (starbase)^51^ was used to study the quantitative expression profiles and the correlation analysis of the genes based on the analysis of TCGA RNA-seq data. As such, the differential expression of miR-23a and the predicted WBP2 CDS-targeting miRNAs between normal and breast invasive carcinoma TCGA samples was analyzed by ENCORI. The RNA-seq data of the same set of clinical samples was analyzed to assess the correlation between WBP2 and MST1 or MST2 or miR-23a. cBioPortal was used to study the survival of the clinical breast cancer samples by splitting the patients with or without harboring genomic alterations of MST1, MST2, WBP2 and miR-23a genes. The two patient cohorts were then compared by a Kaplan–Meier survival plot and log-rank *P*-value was calculated.

### Statistical analyses

All in vitro experiments were performed in three independent experiments. Differences among groups and treatments for all in vitro experiments were determined by two-tailed Student’s *t* test unless otherwise stated. Bar graph represents quantification as mean ± SEM of three biological replicates (*n* = 3). The indicated significance *P* values correspond to <0.05 (∗), <0.01 (∗∗), and <0.001 (∗∗∗).

## Supplementary information

Supplementary Info

Figure S1

Figure S2

Figure S3

Figure S4

Supplementary Table

Supplementary Table S1

Supplementary Table S2

Supplementary Table S3
