# Supplementary material for: Hippo/MST blocks breast cancer by downregulating WBP2 oncogene expression via miRNA processor Dicer
Source: Cell Death Dis. 2020 Aug 21;11(8):669. doi: 10.1038/s41419-020-02901-3 (PMC7441404; doi:10.1038/s41419-020-02901-3)
Supplement: Supplementary file 1 — Supplementary Info [file 41419_2020_2901_MOESM1_ESM.docx]

**Supplementary Materials and Methods**

**Antibodies**

Anti-YAP (#4912), anti-MST1 (#3682), anti-MST2 (#3952), anti-LATS1 (#9153), anti-LATS2 (#5888), anti-phospho-MST1 (Thr183)/MST2 (Thr180) (#3681), anti-phospho-MOB (Thr35) (#8699), anti-phospho-LATS1 (Thr1079) (#8654), anti-SAV (#3507), anti-phospho-YAP (Ser127) (#4911) rabbit polyclonal antibodies were obtained from Cell Signaling Technology Inc. (Danvers, MA, USA); anti-Dicer (sc-136980) mouse monoclonal antibodies were obtained from Santa Cruz Biotechnology (Santa Cruz, CA, USA); Anti-ITCH (#611199) mouse monoclonal antibodies were obtained from BD Biosciences (San Diego, CA, USA); anti-V5 (#MA5-15253), anti-FLAG (#MA1-91878), anti-HA (#26183), anti-Myc (#MA1-980), anti-GADPH (#MA5-15738), anti-β-Tubulin (#MA5-16308) mouse monoclonal antibodies and anti-mouse, anti-rabbit, and anti-goat horseradish peroxidase (HRP) conjugates were obtained from Thermo Fisher Scientific Pierce (Rockford, IL, USA).

**Plasmids and reporters**

The following were purchased from Addgene: Myc-Dicer, Super 8X TOPFlash reporter, pLenti-CMV-Puro/Hygro and pLenti-CMV/TO Puro Dest (Inducible System). Myc-MST1, Myc-MST2, Flag-LATS1, Flag-LATS2, HA-SAV, HA-MOB, Flag-YAP was from Chan Siew Wee (IMCB, A-star, Singapore); pRL-TK was purchased from Promega (Madison, WI, USA). Myc-Dicer catalytic domain mutant, Myc-MST1-K59R and Myc-MST2-K56R were generated using the QuikChange Lightning Site-Directed Mutagenesis Kit (Stratagene; Agilent Technologies, Santa Clara, CA, USA). V5-WBP2 was subcloned in pCDNA6.2 and pLenti-CMV-Puro plasmid. Myc-MST1 and Myc-MST2 were subcloned into the inducible pLenti-CMV/TO Puro Dest and pLenti-CMV-Hygro plasmids.

To construct the psiCheck-2 (Promega, Madison, WI, USA) target luciferase reporter plasmid, the CDS+3’UTR of WBP2 was amplified using PCR of cDNA obtained from total RNA of BT-549 cells. The amplified WBP2 CDS+3’UTR was then cloned into the 3’UTR of the Renilla luciferase gene using the XhoI and NotI restriction sites of the psiCheck-2 vector. Target mutations were made to the CDS+3’UTR region of the sequence, which would interact with seed region of miR-23a, using psiCheck-2-WBP2 3’UTR plasmid as the template.

**5’_WBP2_CDS:**CCGCTCGAGATGGCGCTCAACAAGAATCACTCGGAGGGCGGCGGAGTGATCGTCAATAACACCGAGAGCATCCT**A*ATGT*CCT**ATGATCACGTGGAACTCACATTCAATGACATGAAGAACGTGCCAGAAGCCTTCAAAGGGACCAAGAAAGGCACTGTCTACCTTACCCCTTACCGGGTCATCTTTCTGTCCAAGGGCAAGGATGCCATGCAGTCCTTCATGATGCCATTTTATCTCATGAAAG**AC*TGTG*A**GATCAAGCAGCCCGTATTTGGTGCAAACTACATCAAGGGAACAGTGAAGGCGGAAGCGGGAGGTGGCTGGGAAGGCTCTGCTTCCTACAAGTTGACTTTCACGGCAGGGGGCGCCATTGAGTTCGGACAGCGGATGCTCCAGGTGGCATCTCAAGCCTCCAGAGGTGAAGTCCCCAGTGGAGCCTATGGCTACTCTTACATGCCCAGCGGGGCCTATGTCTATCCCCCGCCAGTCGCCAATGGAATGTACCCCTGCCCTCCTGGCTACCCCTATCCACCGCCCCCACCTGAGTTCTATCCAGGACCCCCCATGATGGACGGGGCCATGGGATACGTGCAGCCCCCACCACCGCCCTACCCTGGGCCCATGGAACCTCCGGTCAGCGGCCCCGATGTCCCCTCCACTCCTGCAGCCGAAGCCAAGGCCGCAGAAGCAGCCGCCAGCGCCTATTACAACCCAGGCAATCCTCACAACGTCTACATGCCCACGAGCCAGCCGCCGCCACCTCCCTACTACCCACCGGAAGATAAGAAGACCCAGTAGG_**WBP2_3’UTR:**CCCTCCTGCCTCCCTGCCTCCCACCCTCATCTCTCTACCCTACCCCTCCCATCGGGGCTGTGCTGGGGCTTGGGGAGGGGAGGGGGCGCCTTGTTCTCCCTCCAGGTCTGATCATAAACAATTACCAGGAACTAGCATTGTGGGACATTAGGGCCCCCGGCCTCGGGAGAGGTGCCGCCCAGCTTCCCATGCCAGCCCGGAGCCCACAGTGCTGCCCAGCGTACCTCCCTCACCGTCTGGGGCTCTTCTGGGAGCACGGAGCATCCCCTGTTCCTGTTTCACTCTCAGCTTCTCCCCTCGAAGGGACTCTCTGGCCACCTCCTCCACCGCAGTCCAGCTCCCTCAGTCTGGCACCCACTGCTACACTCAGCCTCATGAGCCACTTCAGACCAGCCAGGTGTCTTCCCGGGCCCTGCCAGACCCTGCTCACATTCCCTCTGCTGGTCTGTGCTGGTCTCAGAAGGCCACCGCGCCCGCATTCCACTCAGCCAGGGTCCAGCTGCAGCCCCCGCCACCCTTCCTTCCCTTCCCTGTCCTGGGTCATGTTGTTGCCACCCTGTGTGACTTTTGAAGCTGTAAAATGAGCTTCCAGGGCTTGGGTGGCGTCGGGGCAGGGCCGCCGAGGCTGGGAGGAAGCCCTTCTGCCTTTTGCTGGTGTTTCTGGAATTTGCTTTCCCTCACCTCTCACTTCCTTCTAGAAGGAGCTTCCTGACTGGAACCAGAGAATGCATGTCTGTCCACTTGGTGGCTGCTGGGTGGGGCCGGGAACAAGGGCCCCTGACCCTGTGTGCTGGCCGGGACCTGCCACCAGCCCCCCAGCCTGCTTCTTCCCCTTAAGCTTTGTGCCCCTGGATGCGCTAACATTCACTCTTGTTTGTCCCTGGACTGGCCATGAAGTGAGGAGATGGTTATTTAAAGAGAATTCCCTATTTATTTGACAAAAAATCCAGTTAATATATT**AAT*GTGA***AATAAACCCTGTTTGCACCTCGATTTGTTTGCTGAA**AAT*GTGA***AATAGTAAAAATGAAATAACTGGAAAAAAGCGGCCGCAAAAGGAAAA_**3’**

Predicted miR-23a seed binding sites (sites 1-4) on WBP2 mRNA are shown in highlighted boxes. Site-directed mutagenesis was used to mutate each target site. The nucleotides in red fonts were mutated using the following primers:

**Site1 Mutagenesis (ATGT🡪 CCTG):**

WBP2-Site 1-Primer 1: cgtcaataacaccgagagcatccta***cctg***cctatgatcacgtggaactca

WBP2-Site 1-Primer 2: tgagttccacgtgatcatagg***cagg***taggatgctctcggtgttattgacg

| **Length (nt.)** | **Tm** | **Duplex Energy at 68 °C** |
| --- | --- | --- |
| 50 | 78.04℃ | -62.84 kcal/mole |
| 50 | 78.04℃ | -64.53 kcal/mole |

**Site2 Mutagenesis (TGTG 🡪 CACA):**

WBP2-Site 2-Primer 1: gatgccattttatctcatgaaagac***caca***agatcaagcagcccgtatttggtgc

WBP2-Site 2-Primer 2: gcaccaaatacgggctgcttgatct***tgtg***gtctttcatgagataaaatggcatc

| **Length (nt.)** | **Tm** | **Duplex Energy at 68 °C** |
| --- | --- | --- |
| 54 | 78.30℃ | -62.84 kcal/mole |
| 54 | 78.30℃ | -64.53 kcal/mole |

**Site3 Mutagenesis (GTGA 🡪 GCGC):**

WBP2-Site 3-Primer 1: tgacaaaaaatccagttaatatattaat*g****c****g****c***aataaaccctgtttgcacctcg

WBP2-Site 3-Primer 2: cgaggtgcaaacagggtttatt***gcgc***attaatatattaactggattttttgtca

| **Length (nt.)** | **Tm** | **Duplex Energy at 68 °C** |
| --- | --- | --- |
| 54 | 78.02℃ | -62.84 kcal/mole |
| 54 | 78.02℃ | -64.53 kcal/mole |

**Site4 Mutagenesis (GTGA 🡪 GCGC):**

WBP2-Site 4-Primer 1: gcacctcgatttgtttgctgaaaat*g****c****g****c***aatagtaaaaatgaaataactggaaa

WBP2-Site 4-Primer 2: tttccagttatttcatttttactatt***gcgc***attttcagcaaacaaatcgaggtgc

| **Length (nt.)** | **Tm** | **Duplex Energy at 68 °C** |
| --- | --- | --- |
| 55 | 78.32℃ | -62.84 kcal/mole |
| 55 | 78.32℃ | -64.53 kcal/mole |

**siRNA, shRNA and miRNA sequences**

| **Gene** | **siRNA/shRNA** | **Sequence** |
| --- | --- | --- |
| ***WBP2** | siRNA-1 | 5’-CAGGAACUAGCAUUGUGGGACAUUA-3’ |
|  | siRNA-2 | 5’-CCUGGAUGCGCUAACAUUCACUCUU-3’ |
|  | Scramble siRNA | 5’-CAGUCACGAUUAGUGAGGACGAUUA-3’ |
| ***ITCH** | siRNA-1 | 5’-CAAUUUGUCUUGAUGGGCUACAGUU-3’ |
|  | siRNA-2 | 5’-AACUGUAGCCCAUCAAGACAAAUUG-3’ |
| ***Luciferase** | siRNA | 5’-CGUACGCGGAAUACUUCGA-3’ |
| ****MST1** | siRNA | 5’-GGGGUAGCAGGUCAACUUACAGATA-3’ |
| ***MST1** | siRNA-1 | 5’-GACAGCCCUCAUGUAGUCAAAUAUU-3’ |
|  | siRNA-2 | 5’-GGCUGGUUCUGUAUCUGAUAUCAUU-3’ |
|  | siRNA-3 | 5’-GGAGUGUCAAUACUGCGAGACUUAA-3’ |
| ****MST2** | siRNA | 5’-GGAACAGCAACGAGAAUUGGAAGAG-3’ |
| ***MST2** | siRNA-1 | 5’-CCUGUUGAAUCAGAUCUUCAGGAAA-3’ |
|  | siRNA-2 | 5’-GGUCAGUUAACAGAUACAAUGGCAA-3’ |
|  | siRNA-3 | 5’-CAAGAAUGCCAAACCUGUAUCAAUA-3’ |
| ****LATS1** | siRNA-1 | 5’-CCCAUAUAAUUAUCCGAAGCCUATT-3 |
|  | siRNA-2 | 5’-GAAGGAUAUAGACAAAUGAGGCCTA-3’ |
| **Dicer** | *siRNA-1 | 5’-CCAGCACUUUGGAUAUUGACUUUAA-3’ |
|  | **siRNA-2 | 5’-CGAGUUAGACAUAUGAAAUGGUUAT-3’ |
| *****miR-19a** | miRNA mimic | 5’-UGUGCAAAUCUAUGCAAAACUGA-3’ |
|  | miRNA inhibitor | 5’-UGUGCAAAUCUAUGCAAAACUGA-3’ |
| *****miR-19b** | miRNA mimic | 5’-UGUGCAAAUCCAUGCAAAACUGA-3’ |
|  | miRNA inhibitor | 5’-UGUGCAAAUCCAUGCAAAACUGA-3’ |
| *****miR-23a** | miRNA mimic | 5’-AUCACAUUGCCAGGGAUUUCC-3’ |
|  | miRNA inhibitor | 5’-GGAAAUCCCUGGCAAUGUGAU-3’ |
| ****Universal Negative Ctrl** | siRNA | 5’-AUACGCGUAUUAUACGCGAUUAACGAC-3’ |

*siRNAs were obtained from Thermo Fisher Scientific Invitrogen (Carlsbad, California, USA). **siRNA were obtained from IDT (Coralville, IA, USA).

***miRNA mimic/inhibitor and TAZ ON-TARGETplus siRNA were obtained from GE Dharmacon (Lafayette, CO, USA).

**Cell culture**

MCF10A, MCF12A, MCF12F, HMEC, MDA-MB453, BT474, ZR-75-1, MCF7, MDA-MB231, T47D, SK-BR3, MDA-MB468, BT549, BT20, HS578T, MDA-MB361, MDA-MB436 and HeLa cells were obtained from American Type Culture Collection (Manassas, VA, USA). MCF7, ZR-75-1, BT474, BT549, T47D, HeLa, and MDA-MB231 cells were maintained in RPMI-1640 (Thermo Scientific Hyclone, South Logan, UT, USA) containing 10% FBS (Hyclone) and 100U penicillin/streptomycin (Biological Industries, Israel). BT20, MDA-MB453, MDA-MB361, SK-BR3, MDA-MB468, HS578T, MDA-MB436 cells were maintained in DMEM (Hyclone) containing 10% FBS and 100U penicillin/streptomycin. MCF10A, MCF12A and MCF12F cells were maintained in DMEM/F12 (Sigma) containing 5% horse serum with additives previously described ^1^. HMEC cells were cultured in HuMEC Ready Medium (Gibco). Unless otherwise stated, cell lines were mostly obtained between year 2005 to 2011 from ATCC, which authenticates using short tandem repeat profiling. They are expanded upon receipt, frozen in multiple vials and maintained by passaging not exceeding one month. The WNT3A conditioned medium was prepared as directed by ATCC.

**Transient transfection and stable selection**

Cells were transiently reversed transfected using jetPRIME reagent (Polyplus Transfection, Illkirch, France), according to manufacturer's instructions and harvested at 24–48 h post-transfection.

For stable inducible expression, a T-REX line of MDA-MB231 and MDA-MB468 were first generated, followed by a second round of clonal selection for 2-3 weeks on T-REX line transfected with WBP2 inducible mammalian expression plasmid. Selected clones were screened for inducible MST1/2 protein overexpression upon treatment with 1 ug/ml doxycycline for 3 days. Positive clones were expanded and maintained with puromycin. MST1/2 expression was checked periodically.

MDA-MB436 cells were transduced with WBP2-expressing lentivirus particles. At 48 hours post-infection, 2×105 cells in 100-mm dish were exposed to 0.5 µg/ml puromycin (Gibco) for 3 weeks and screened for WBP2 protein overexpression. Selected positive clones were pooled and maintained with same selection pressure. WBP2 expression was checked periodically.

**Lentivirus production and transduction**

The 293FT cells (Thermo Fisher Scientific) were used for lentivirus production and regularly maintained in DMEM medium supplemented with 0.5 mg/ml G418 (Invitrogen). The 293FT cells were transiently transfected with WBP2-pLenti-CMV-Puro or MST1/2-pLenti-CMV-Hygro plasmid and 3 other packaging plasmids: pRSV-Rev, pMDLg/pRRE and pCMV-VSVG in the ratio of 5:2:2:1 and replenished with fresh medium 6-8 hours post-transfection. The supernatant containing lentiviral particles was collected at 48-72 hours post-transfection, cleared by centrifugation at 1,250 rpm for 5 minutes and filtrated through a 0.45 µm low protein binding membrane. One day before lentivirus transduction, cells were seeded at 60-70% confluency in 6-well plates. Collected virus supernatant supplemented with 4µg/mL polybrene (Sigma) was then added to the cells and the plates were spun at 1,000 g for 2 hours at room temperature with a swing bucket centrifuge. After centrifugation, the cells were further incubated with the virus supernatant for another 2 hours before replacing with fresh medium. 48 hours post-infection, the cells were harvested.

**Cell lysis and immunoblotting**

Cells were washed twice with ice-cold PBS and lysed in NID (Non-Ionic Detergent) lysis buffer (50 mM Tris, pH7.4, 150 mM NaCl, 1 mM EDTA, 0.5% Nonidet-P40, 0.5% Triton X-100) containing protease and phosphatase inhibitor cocktail (Pierce). Cell lysates were vortexed and cleared by centrifugation at 16000 g for 10 min at 4 °C. The protein concentration of the lysates was assayed using the BradfordUltra Protein Assay reagent (Expedeon, San Diego, CA, USA). 50 µg proteins were resolved by SDS-PAGE on 8-12% gels. The proteins were subsequently transferred on to PVDF membranes (Pierce). After incubation in blocking buffer [5% (w/v) non-fat dried milk in TBST or BSA], the blots were incubated with various antibodies at their optimal dilutions overnight at 4 °C. The goat anti-rabbit/mouse HRP-conjugated secondary antibodies (Pierce) were diluted at 1:10000 in blocking buffer, incubated with the blot for 1 h at room temperature and detected by Pierce ECL Western Blotting or Advansta WesternBright ECL HRP (Menlo Park, CA, USA) substrate. In all panels, experiments were repeated 2-3 times and representative immunoblots shown.

**Gene Expression Analysis**

Total RNA was isolated using Quick-RNA Kit (Zymo Research) and reversed transcribed with RevertAid First Strand cDNA Synthesis Kit (Thermo Scientific) per manufacturer’s instructions. Total miRNA was isolated using mirVana miRNA Isolation Kit and reversed transcribed with TaqMan MicroRNA Reverse Transcription Kit per manufacturer’s instructions. The transcript and miRNA abundance was assayed by qRT-PCR using QuantiFast Probe PCR kit (Qiagen, Venlo, Netherlands) and TaqMan MicroRNA Assay kit respectively, as recommended. Pre-designed primers/probes were obtained from IDT (Coralville, Iowa, USA) and available upon request.

**Supplementary Figure Legends**

**Fig. S1**

**A:** (i) qPCR analysis and IB analysis of WNT3A/WBP2-induced Wnt target gene-AXIN2 mRNA (i) and protein (ii) expression in HeLa cells transiently transfected with MST1/2-WT+SAV. **B:** (i) qPCR analysis of LATS1 and LATS2 mRNA expression in HeLa cells transfected with vector/MST1+2-SAV and scrambled/LATS1+2 siRNAs. (ii) Quantitative densitometry analysis of LATS1/2-MOB-mediated WBP2 downregulation upon MST1/2 kncokdown in HeLa (**Fig. 1D-ii**).

**C:** IB analysis of endogenous WBP2 in MB231 T-Rex cells stably infected with vector or MST1 or MST2 upon doxycycline treatment for 72 hours.

**D:** IB analysis of phosphorylation of endogenous MST1/2 in MST1 or MST2-transiently transfected MB231 cells upon doxycycline treatment for 72 hours.

**E:** Correlation analysis of WBP2 and MST1 or MST2 protein expression in a panel of breast cancer cell lines via linear regression plots to determine the degree of correlation between WBP2 and MST protein expression.

**F:** IB analysis of MOB phosphorylation upon doxycycline-induced MST1/2 expression in stable MDA-MB-231 (i) or MDA-MB-468 (ii) T-Rex cells.

**Fig. S2**

**A:** Vector- and WBP2-WT-overexpressing HeLa cells were transfected with SAV and MST1-K59R or MST1-WT for 24 hours before being seeded for *in vitro* growth analysis in soft agar assay. Only colonies >100 µM were scored.

**B:** WBP2-WT-overexpressing HeLa cells were transfected with SAV and vector or MST1-K59R or MST1-WT for 24 hours before being harvested for tumor xenograft study. The *in vivo* growth of xenografted tumors were monitored up to 14 days. Time course line plot (i), dot plot (ii) and images of individual tumors (iii) were shown.

**C:** MB436 cells were transfected with vector, WBP2 and/or MST1-K59R for 24 hours before being seeded in ultra-low attachment plate for analysis of their *in vitro* 3D proliferations after 5 days.

**Fig. S3**

**A:** ITCH knockdown failed to rescue the MST1/2-SAV overexpression (i) or OA (ii) -induced downregulation of WBP2 in HeLa or MB231 and MB468 respectively.

**B:** Inhibition of proteasome (MG132/Lactacystin) or lysosome (Concanamycin A/Chloroquine) did not rescue the MST1/2-SAV overexpression (i) or OA (ii) -induced downregulation of WBP2 in HeLa or MB231 and MB468 respectively.

**C:** Effect of TAZ-WT (i) or YAP-WT (ii) overexpression on MST1-induced WBP2 downregulation.

**Fig. S4**

**A-B**: Dicer (A) or MST1+2 (B) knockdown by another distinct set of siRNA sequences led to upregulation of endogenous WBP2 and/or downregulation of endogenous Dicer in BT549.

**C:** Overexpression of DGCR8 led to downregulation of endogenous WBP2 in BT549.

**D:** The in-frame deletion mutant of Dicer (mutDicer) lost its catalytic activity of miRNA processing.

**E:** Dicer overexpression has a stronger effect on downregulation of endogenous WBP2 compared to miR-23a mimic overexpression.

**F:** miR-23a inhibitor partially rescues the Dicer overexpression-mediated WBP2 down-regulation.

**G:** IB analysis of exogenously expressed WBP2 upon overexpression of WBP2 CDS-targeting miRNAs in BT-549.

**H:** Dicer overexpression specifically downregulates exogenously expressed WBP2, but not NRD or p47.

**I:** TCGA data analysis showing down-regulation of miR-23a in breast invasive carcinoma samples.

**J:** IB analysis of WBP2-WT and its various Ser/Thr mutants upon MST1/2 overexpression in HeLa. Predicted Ser/Thr sites on WBP2 targeted by MST1 (Ser107), MST2 (Ser146, Ser245, Thr260) and MST1/2 (Ser20, Ser131, Thr31, Thr45, Thr49) were identified using GPS 3.0 Prediction.

**Supp Table**

**Table S1:** The predicted list of WBP2-targeting miRNAs using different publicly available online algorithms. Only the miRNAs with sum score ≥ 6 are shown.

**Table S2:** Sum score comparison of miR-23a against known WBP2 3’UTR-targeting siRNAs.

**Table S3:** The prediction list of high-score WBP2 CDS-targeting miRNAs.
