# Supplementary figures and images for: Hippo/MST blocks breast cancer by downregulating WBP2 oncogene expression via miRNA processor Dicer
Source: Cell Death Dis. 2020 Aug 21;11(8):669. doi: 10.1038/s41419-020-02901-3 (PMC7441404; doi:10.1038/s41419-020-02901-3)

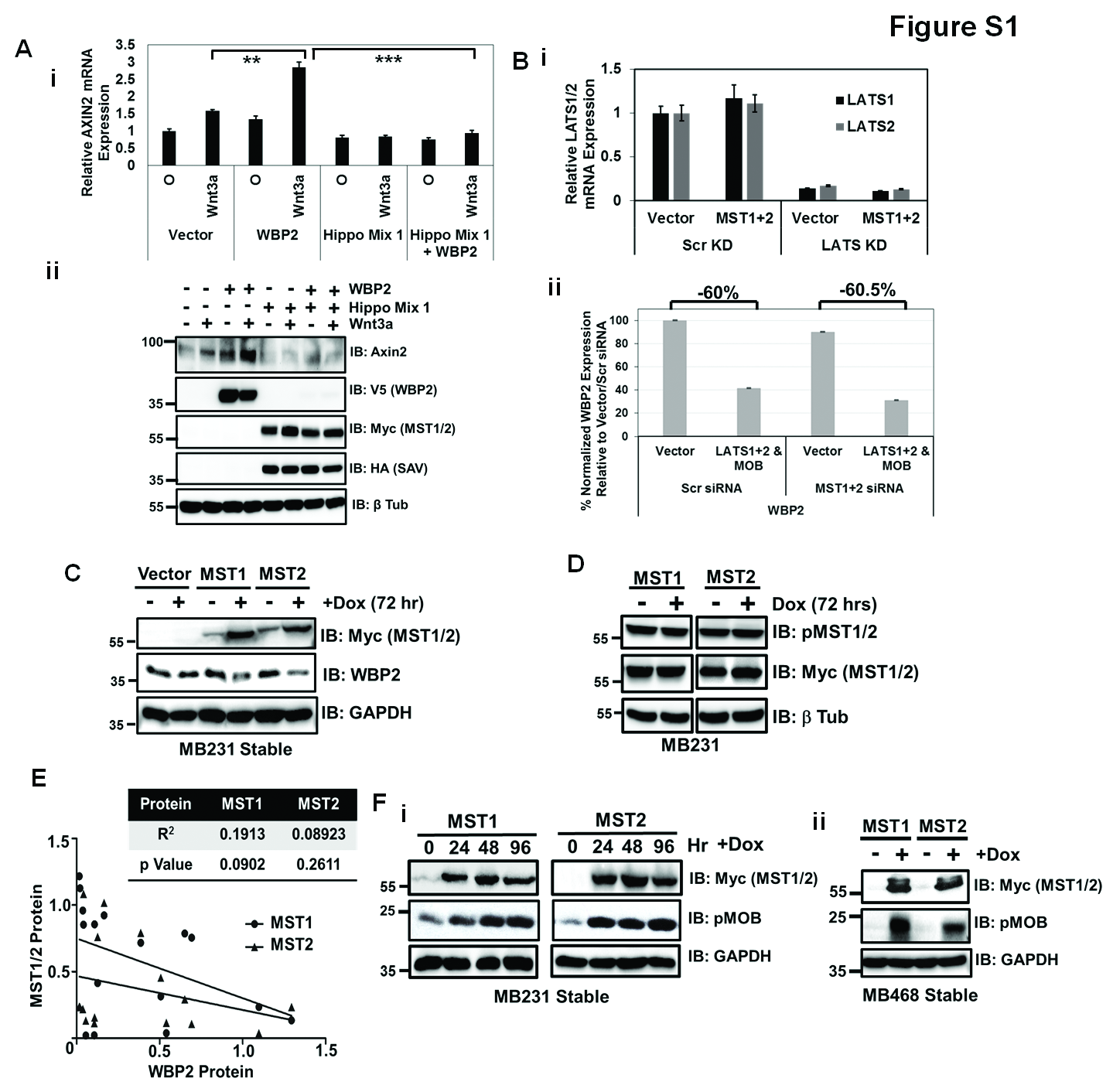

Supplement: Supplementary file 2 — Figure S1 [file 41419_2020_2901_MOESM2_ESM.tif]

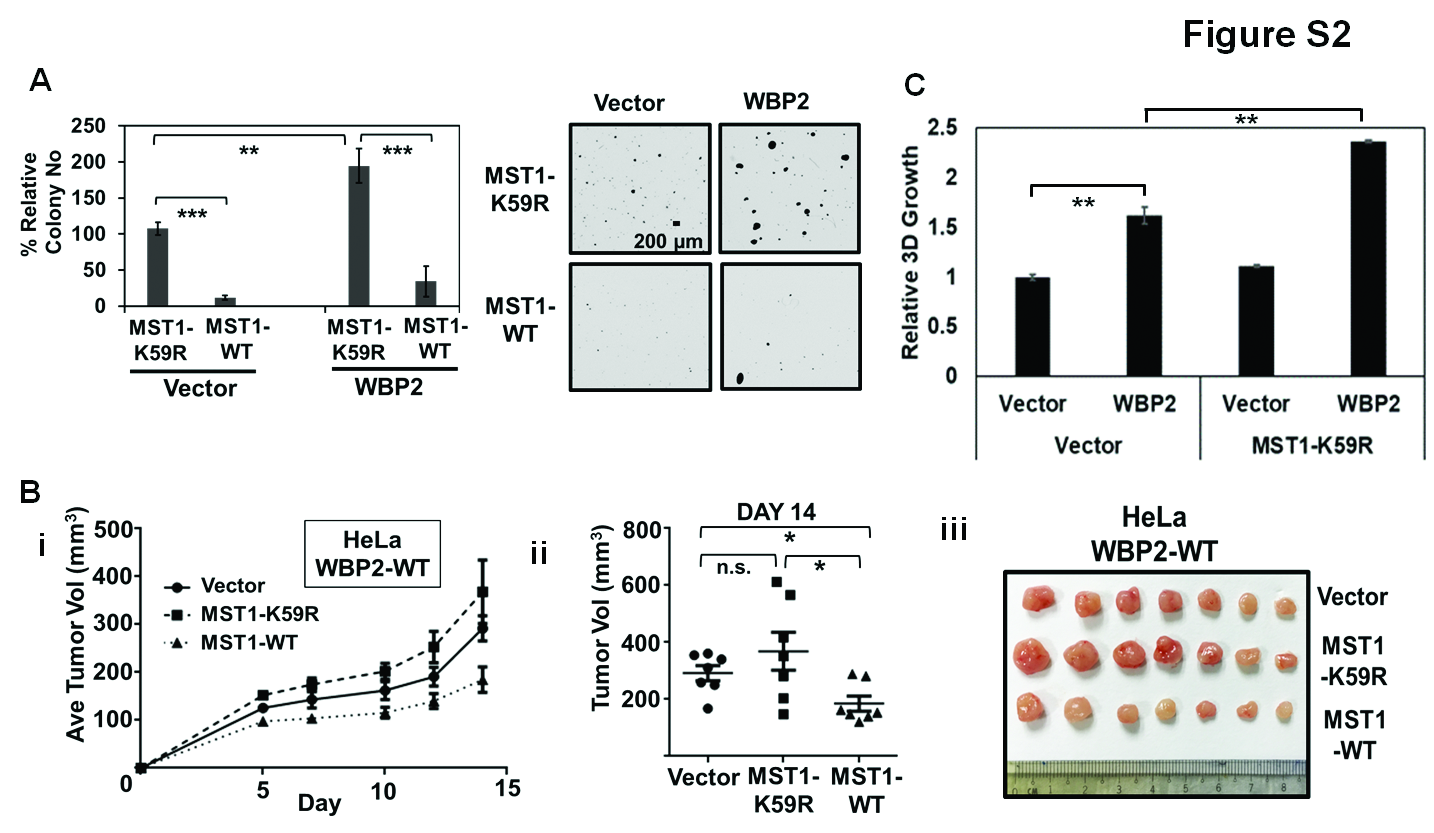

Supplement: Supplementary file 3 — Figure S2 [file 41419_2020_2901_MOESM3_ESM.tif]

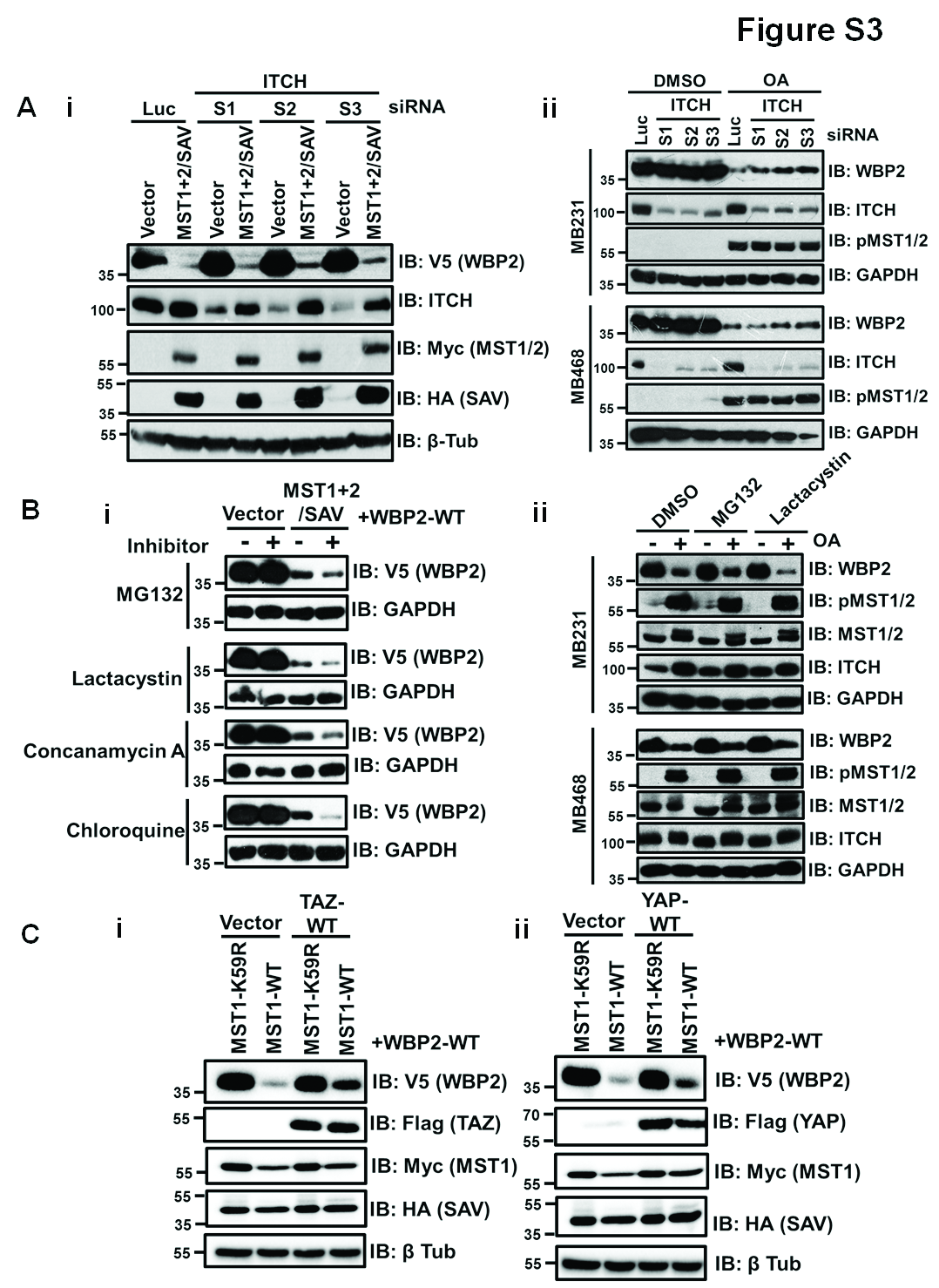

Supplement: Supplementary file 4 — Figure S3 [file 41419_2020_2901_MOESM4_ESM.tif]

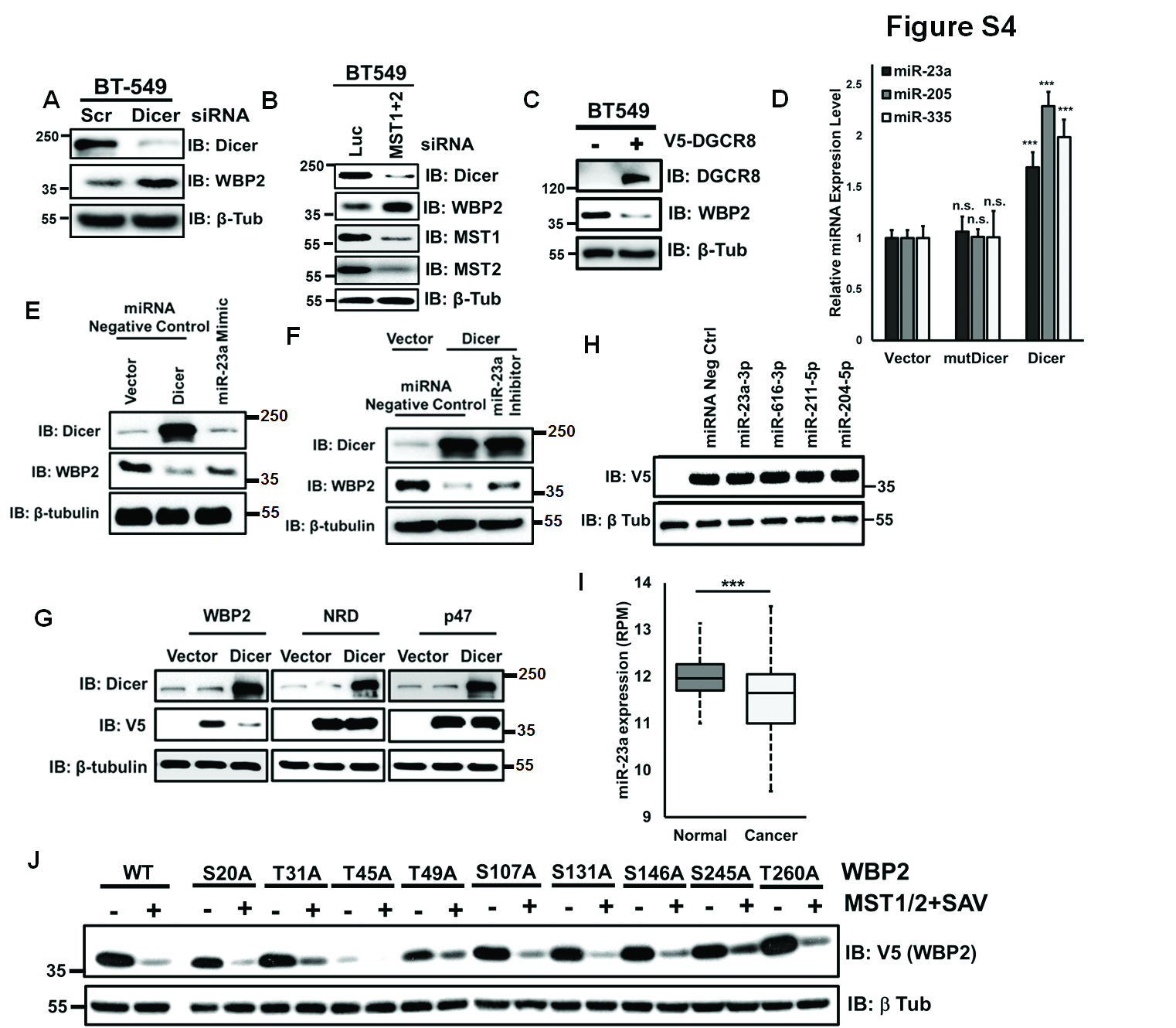

Supplement: Supplementary file 5 — Figure S4 [file 41419_2020_2901_MOESM5_ESM.tif]

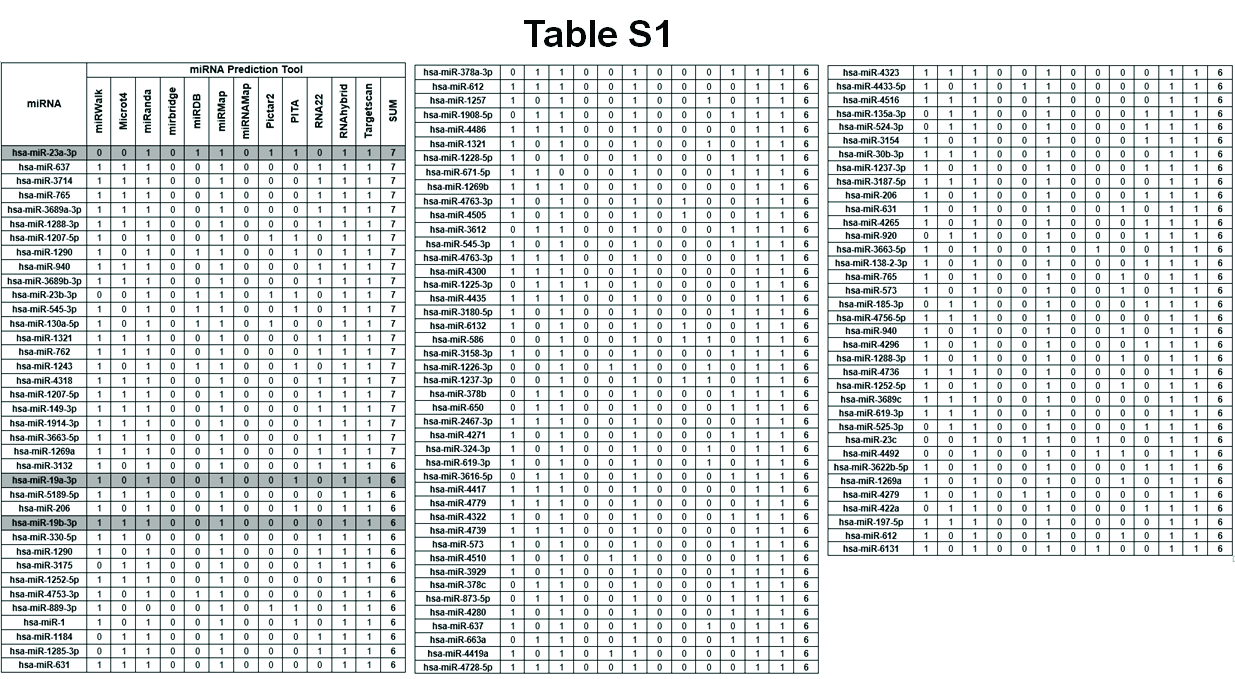

Supplement: Supplementary file 7 — Supplementary Table S1 [file 41419_2020_2901_MOESM7_ESM.tif]

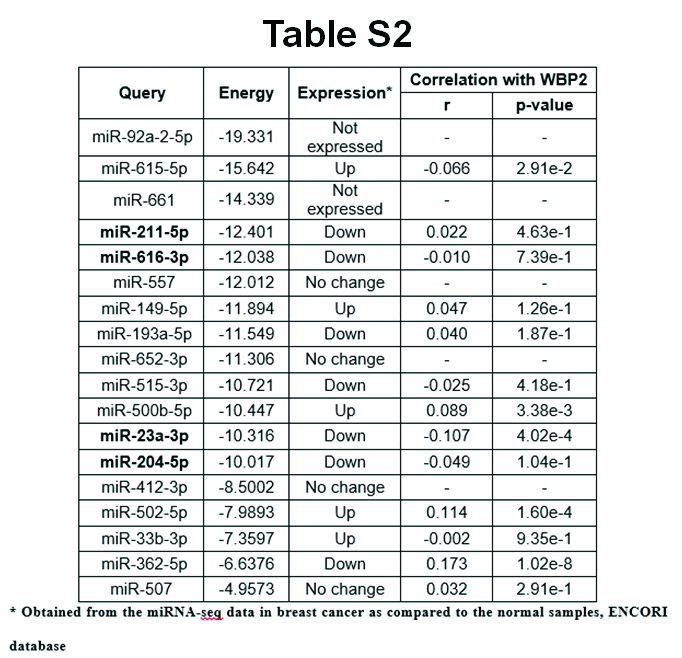

Supplement: Supplementary file 8 — Supplementary Table S2 [file 41419_2020_2901_MOESM8_ESM.tif]

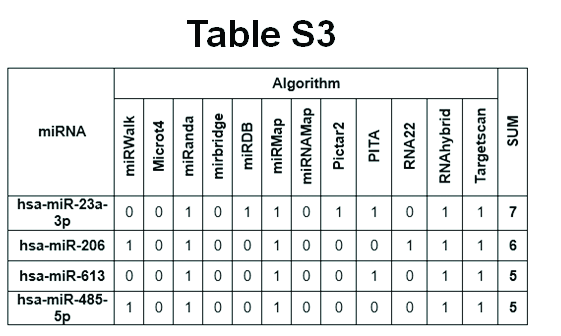

Supplement: Supplementary file 9 — Supplementary Table S3 [file 41419_2020_2901_MOESM9_ESM.tif]
